# Identity-by-descent detection across 487,409 British samples reveals fine scale population structure and ultra-rare variant associations

**DOI:** 10.1038/s41467-020-19588-x

**Published:** 2020-11-30

**Authors:** Juba Nait Saada, Georgios Kalantzis, Derek Shyr, Fergus Cooper, Martin Robinson, Alexander Gusev, Pier Francesco Palamara

**Affiliations:** 1 Department of Statistics, University of Oxford, Oxford, UK; 2 Department of Biostatistics, Harvard T.H. Chan School of Public Health, Boston, MA 02115 USA; 3 Department of Computer Science, University of Oxford, Oxford, UK; 4 Brigham & Women’s Hospital, Division of Genetics, Boston, MA 02215 USA; 5 Department of Medical Oncology, Dana-Farber Cancer Institute, Boston, MA 02215 USA; 6 Wellcome Centre for Human Genetics, University of Oxford, Oxford, UK

**Keywords:** Genome-wide association studies, Haplotypes, Heritable quantitative trait, Population genetics

## Abstract

Detection of Identical-By-Descent (IBD) segments provides a fundamental measure of genetic relatedness and plays a key role in a wide range of analyses. We develop FastSMC, an IBD detection algorithm that combines a fast heuristic search with accurate coalescent-based likelihood calculations. FastSMC enables biobank-scale detection and dating of IBD segments within several thousands of years in the past. We apply FastSMC to 487,409 UK Biobank samples and detect ~214 billion IBD segments transmitted by shared ancestors within the past 1500 years, obtaining a fine-grained picture of genetic relatedness in the UK. Sharing of common ancestors strongly correlates with geographic distance, enabling the use of genomic data to localize a sample’s birth coordinates with a median error of 45 km. We seek evidence of recent positive selection by identifying loci with unusually strong shared ancestry and detect 12 genome-wide significant signals. We devise an IBD-based test for association between phenotype and ultra-rare loss-of-function variation, identifying 29 association signals in 7 blood-related traits.

## Abstract (web summary)

Accurately measuring genetic relatedness by Identical-By-Descent (IBD) segments is challenging in biobank-level genome data. The authors present IBD method FastSMC, which when applied to the UK Biobank gives a detailed picture of genetic relatedness and evolutionary history in the UK over the past 2000 years.

## Introduction

Large-scale genomic collection, through efforts like the NIH All of Us research program^1^, the UK BioBank^2^, Genomics England^3^, and the Million Veteran Program^4^, is expected to yield datasets of hundreds of thousands of individuals and to grow to millions in the coming years. Utilizing such datasets to understand disease and health outcomes requires understanding the fine-scale genetic relationships between individuals. These relationships can be characterized using short segments (less than 10 centimorgans [cM] in length) that are inherited identical by descent (IBD) from a common ancestor between purportedly unrelated pairs of individuals in a dataset^5^. Accurate detection of shared IBD segments has a number of downstream applications, which include reconstructing the fine-scale demographic history of a population^6–9^, detecting signatures of recent adaptation^10,11^, discovering phenotypic association^12,13^, estimating haplotype phase^5,14,15^, and imputing missing genotype data^16,17^, a key step in genome-wide association studies (GWAS)^18^. Detection of IBD segments in millions of individuals within modern biobanks poses a number of computational challenges. Although several IBD detection methods have been published^19–21^, few scale to analyses comprising more than several thousand samples. However, scalable methods that do exist trade modeling accuracy for computational speed. As a result, current IBD detection algorithms are either scalable but heuristic, solely relying on genotypic similarity to detect shared ancestry and not providing calibrated estimates of uncertainty, or too slow to be applied to modern biobanks. Here, we introduce an IBD detection algorithm, called fast sequentially Markovian coalescent (FastSMC), which is both fast, enabling IBD analysis of modern biobank datasets, and accurate, relying on coalescence modeling to detect short IBD segments (down to 0.1 cM). FastSMC quantifies uncertainty and estimates the time to most recent common ancestor (TMRCA) for individuals that share IBD segments. It does so by efficiently leveraging information provided by allele sharing, genotype frequencies, and demographic history, which results in a cost-effective boost in accuracy.

We validate the scalability, accuracy, and robustness of the FastSMC algorithm in detecting IBD sharing within recent millennia using extensive coalescent simulation. Leveraging the speed and accuracy of FastSMC, we analyze IBD sharing in 487, 409 phased individuals from the UK Biobank dataset, identifying and characterizing  ~214 billion IBD segments transmitted by shared ancestors within the past 50 generations. This network of shared ancestry enables us to reconstruct a fine-grained picture of time-dependent genomic relatedness in the UK. Analysis of the distribution of recent sharing within specific genomic regions reveals evidence of recent positive selection at 12 loci. We find the sharing of IBD to be highly correlated with geographic distance and the sharing of rare variants. Leveraging this correlation, we detect 20 associations to genomic loci harboring loss-of-function (LoF) variants with seven blood-related phenotypes. These results underscore the importance of modeling distant relatedness to reveal subtle population structure, recent evolutionary history, and rare pathogenic variation.

## Results

### Overview of the FastSMC method

The algorithm we developed, called FastSMC, detects IBD segments using a two-step procedure. In the first step (identification), FastSMC uses genotype hashing to rapidly identify IBD candidate segments, which enables us to scale to very large datasets. In the second step (verification), each candidate segment is tested using a coalescent hidden Markov model (HMM), which enables us to improve accuracy, compute the posterior probability that the segment is IBD (IBD quality score), and provide an estimate for the TMRCA in the genomic region. The identification step leverages the GERMLINE2 algorithm that we developed and present here, which improves over GERMLINE’s^19^ speed and memory requirements and thus enables us to very efficiently detect IBD candidate regions in millions of genotyped samples. GERMLINE2 utilizes hash functions to identify pairs of individuals whose genomes are identical in small genomic regions, thus being Identical-By-State (IBS). The presence of these short identical segments triggers a local search for longer segments that are likely to reflect recent TMRCA and thus IBD sharing in the region. Although the original GERMLINE algorithm utilizes a similar strategy, GERMLINE2 offers two key improvements, which result in faster computation and lower memory consumption. First, the GERMLINE algorithm can become inefficient in regions where certain short haplotypes can be extremely common in the population (e.g. due to high linkage disequilibrium), which results in hash collisions across a large fraction of samples, effectively reverting back to a nearly all-pairs analysis and monopolizing computation time. GERMLINE2 avoids this issue by introducing recursive hash tables, which require haplotypes to be sufficiently diverse before they are explored for pairwise analysis and significantly decrease downstream computation (Supplementary Fig. 1). Second, the GERMLINE local search (extension) step requires storing the entire genotype dataset in memory, which is prohibitive for biobank-scale analyses. Instead, GERMLINE2 uses an on-line strategy, reading a polymorphic site at a time without storing complete genotype information in memory, which enables scaling this analysis to millions of individuals. While long IBS regions are often co-inherited from recent common ancestors, thus being IBD, this need not always be the case^22^. In its verification step, FastSMC thus leverages coalescence modeling to filter out candidate segments that are IBS, but not IBD. To achieve this, FastSMC analyzes every detected candidate segment using the ASMC algorithm^23^, a recently proposed coalescent-based HMM that builds on recent advances in population genetics inference^24–27^ to enable efficient estimation of the posterior of the TMRCA for a pair of individuals at each site along the genome. A key advantage of the ASMC algorithm over previous coalescent-based models is that it enables estimating the TMRCA in SNP array data in addition to sequencing data. FastSMC can thus be tuned to be applied to both types of data. FastSMC produces a list of pairwise IBD segments with each segment associated to an IBD quality score - i.e the average probability of the TMRCA being between present time and the user-specified time threshold – and an age estimate – i.e. the average maximum a posteriori (MAP) TMRCA along the segment. FastSMC can be parallelized to efficiently scale up to biobank datasets. The FastSMC software implements both the GERMLINE2 and ASMC algorithms, and is freely available.

### Comparison to existing methods

**Fig. 1:**
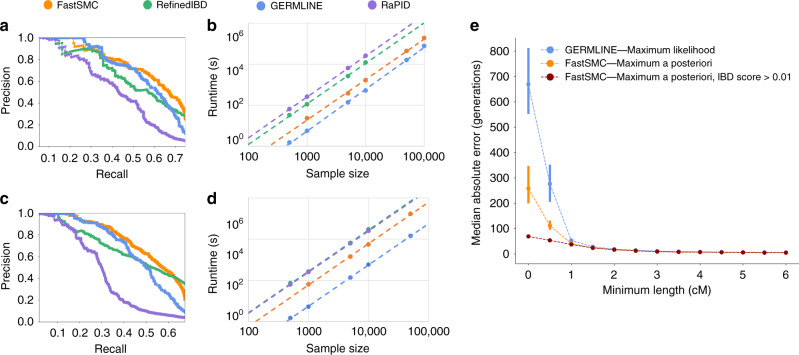
FastSMC in coalescent simulations. **a** (respectively **c**). Precision-recall curve randomly sampled from 10 realistic European simulated datasets with 300 haploid samples for IBD segments detection within the past 50 generations (respectively 100 generations), within the recall range where all methods are able to provide predictions. **b** (respectively **d**). Running time (CPU seconds) using chromosome 20 of the UK Biobank for IBD segments detection within the past 50 generations (respectively 100 generations). The entire cohort of 487, 409 samples (across 7913 SNPs) was randomly downsampled into smaller batches. Only one thread was used for each method and running time trend lines in logarithmic scale are shown, reflecting differences in the quadratic components of each algorithm. Parameters for all methods were optimized to maximize accuracy and used for both accuracy and running time benchmarking (details in Methods section). **e** Median absolute error of the length-based maximum likelihood age estimate (MLE) on segments detected by GERMLINE in blue and our MAP age estimate on segments detected by FastSMC (no filtering on the IBD quality score in orange, and a minimum IBD quality score of 0.01 in dark red) for 300 haploid simulated samples with European ancestry. As a reference, the average TMRCA at a random site for a pair is in the order of several thousands of generations^23^. Only detected IBD segments longer than the minimum length represented on the *X*-axis were considered. We ran both algorithms with the same minimum length of 0.001 cM and other parameters from grid search results for a time threshold of 50 generations. Data are represented as mean values ± SEM over 10 simulations.

We measured FastSMC’s accuracy using extensive realistic coalescent simulations that mimic data from the UK Biobank^2^. We benchmarked IBD detection for FastSMC in addition to three other widely used or recently published IBD detection methods: GERMLINE^19^, RefinedIBD^20^, and RaPID^21^. Parameters for all methods were optimized to maximize accuracy and evaluated on the detection of IBD segments within the past 25, 50, 100, 150, or 200 generations on simulated populations with a European ancestry (Supplementary Table 1). We found that FastSMC outperforms the accuracy of all the other methods at all time scales (Fig. 1a, c, Supplementary Fig. 2, and Supplementary Table 2). As expected, model-based algorithms such as FastSMC and RefinedIBD tend to achieve better results in detecting older (shorter) IBD segments than genotype-matching methods, which cannot reliably exclude short segments where genotypes are IBS but not IBD (Supplementary Table 2). FastSMC relies on the ASMC algorithm in its validation step, which was shown to be robust to the use of an inaccurate recombination rate map or violations of assumptions on allele frequencies in SNP ascertainment^23^. We thus expect it to be similarly robust to several types of model misspecification. In particular, we tested the effects of using a misspecified demographic model on FastSMC’s accuracy, and observed that while this results in biased estimates of segment age (Supplementary Fig. 3), a wrong demographic model does not affect accuracy (Supplementary Table 3).

Next, we evaluated the computational efficiency of FastSMC and other methods using phased data from chromosome 20 of the UK BioBank (Fig. 1b, d and Supplementary Fig. 4). As expected, the improvement in accuracy achieved leveraging FastSMC’s validation step leads to slightly increased computing time compared to only using GERMLINE, the most scalable method. FastSMC is faster than RefinedIBD, the closest method in terms of accuracy for short segments. For instance, detecting IBD segments within the past 50 generations on 10,000 samples takes 27 min for FastSMC, 9 min for GERMLINE, 3 h and 17 min for RefinedIBD, and 6 h and 58 min for RaPID. We finally assessed the memory cost of FastSMC and other methods (Supplementary Fig. 5). FastSMC does not store the genotype hashing or IBD segments, resulting in a very low memory footprint, whereas the memory requirements of other methods become prohibitive for large sample sizes such as those required to analyze the entire UK Biobank cohort. For example, analyzing chromosome 20 for a time threshold of 50 generations and 10,000 random diploid individuals from the UK Biobank dataset, FastSMC requires 1.4 GB of RAM compared to 3.8 GB for GERMLINE, 11.5 GB for RefinedIBD, and 62.9 GB for RaPID.

Downstream analysis of IBD sharing such as demographic inference or the study of natural selection often involves estimating the age of IBD segments. Because current approaches do not explicitly model the TMRCA between IBD individuals, segment age is typically estimated through the length of the IBD segment. FastSMC, on the other hand, explicitly models TMRCA across individuals, leveraging additional prior information (such as demography and allele frequencies) to produce an improved estimate of IBD segment age. We found FastSMC’s segment age estimates to be more accurate than a length-based estimator (Supplementary Fig. 6), with significant gains for short segments as a result of the additional modeling in the validation step of the algorithm (e.g. the median error from FastSMC’s segments age estimate decreased by  ~60% for segments ≥0.5 cM compared to the current approach, Fig. 1e). FastSMC’s increased accuracy in estimating coalescence times in IBD segments will translate in improved resolution for downstream applications that leverage this type of information.

### IBD sharing and population structure in the United Kingdom

We leveraged the scalability and accuracy of FastSMC to analyze 487,409 phased British samples from the UK Biobank, obtaining a fine-grained picture of the genetic structure of the United Kingdom. We detected  ~214 billion IBD segments shared within the past 1500 years, with ~75% of all pairs of individuals sharing at least one common ancestor (Supplementary Fig. 7). Analyzing the fraction of genome covered by IBD segments, we observed that  ~93% of individuals in the cohort have >90% of their genome covered by at least one IBD segment in the past 50 generations, compared to only  ~4% of individuals for the past 10 generations (Supplementary Fig. 8A). Looking for geographic patterns, we noticed that, despite the large sample size of the UK Biobank cohort, the average fraction of genome covered by at least one IBD segment is substantially heterogeneous across UK postcodes for recent time scales – ranging from 53.4% in London Eastern Central (EC) to 76.2% in Stockport (SK) for 10 generations – but more uniform at deeper time scales – ranging from 96.7% in London EC to 99.2% in Stockport for 50 generations (Supplementary Fig. 8B, C, D). The observation of a non-uniform IBD coverage has implications for downstream methods that rely on distant relatedness at each genomic site, such as variant discovery, phasing, and imputation.

**Fig. 2:**
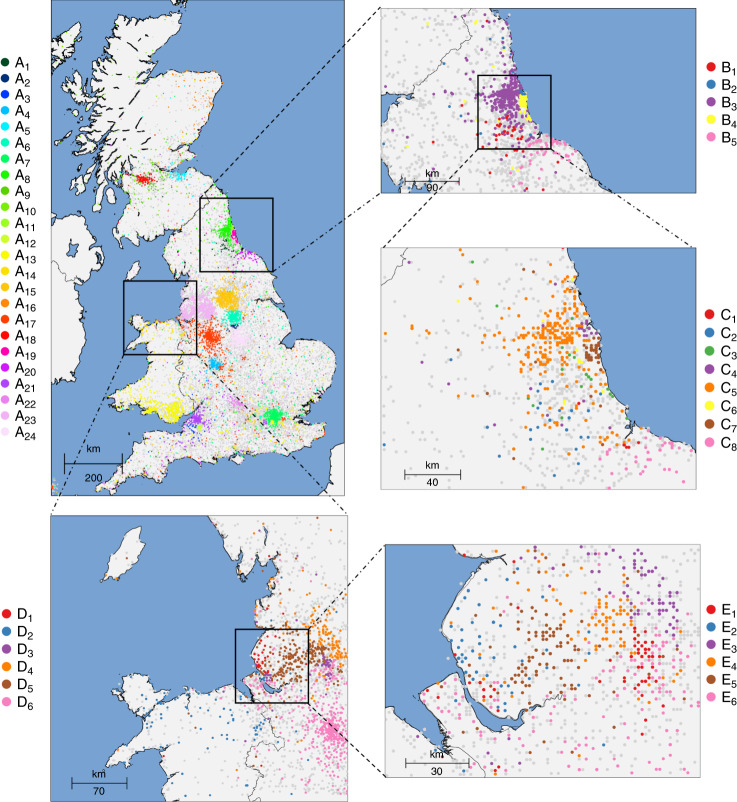
Fine-scale population structure in the UK. Hierarchical clustering of 432,866 individuals from the UK Biobank dataset based on the sharing of IBD segments within the past 10 generations. Individuals in clusters with <500 samples are shown in light gray. We observed 24 main clusters across the country (top left) and we refined two regions, corresponding to Newcastle (NE) (top right) and Liverpool (L) (bottom), revealing fine scale population structure. No relationship between clusters is implied by the colors or cluster labeling across different plots (details are provided in Methods section).

Next, we analyzed the network of recent genetic relatedness for 432,968 samples for whom birth coordinates are available. As observed in previous studies of fine-scale genetic structure^28^ with a considerably smaller sample size^29^, genetic clusters within the UK tend to be localized within geographic regions (Fig. 2). Leveraging FastSMC’s accuracy and the large sample size of the UK Biobank dataset we were able to zoom into increasingly smaller regions, finding that such clusters extend beyond broad geographic clines. Smaller geographic regions revealed increasingly fine-grained clusters of individuals born within a few tens of kilometers from each other, likely reflecting the presence of extended families which experienced limited migration during recent centuries. We found that individuals throughout the UK, including cosmopolitan regions, find overwhelmingly more recent genetic ancestors within their own postcode than in other regions of the country, reflecting isolation-by-distance due to limited migration across the country in recent generations (Supplementary Fig. 9 and https://ukancestrymap.github.io/ for an interactive website displaying these results). For instance, within the past  ~300 years, two individuals born in North London share on average 0.0092 common ancestors and two individuals born in Birmingham share on average 0.0043 common ancestors. In contrast, and despite the relative geographic proximity, an individual born in North London shares on average a substantially lower 0.00059 ancestors with one born in Birmingham. We observed similar trends when we restricted this analysis to samples of self-reported and inferred non-White British ancestry^2^, as shown in Supplementary Fig. 10. We further visualized the strong link between genetic and physical distances (Supplementary Fig. 11) by building a low-dimensional planar representation of pairwise genetic distances across postcodes within the past 600 years (Supplementary Fig. 12B), which we found to closely reflect geographic distance across these regions.

**Fig. 3:**
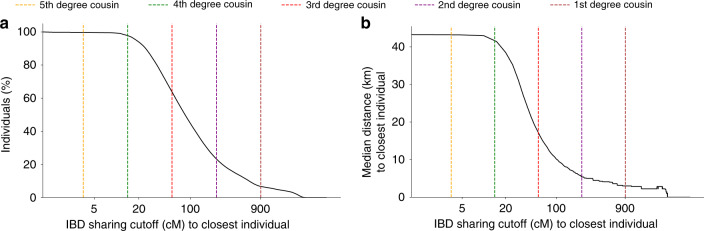
Genetic relatedness and geographic distances in the UK Biobank dataset. For each of the 432,968 UK Biobank samples with available geographic data, we detected the individual sharing the largest total amount (in cM) of genome IBD within the past 10 generations (referred to as closest individual). **a** For each value *x* of total shared genome (in cM) on the *X*-axis, we report the percentage of UK Biobank samples (*Y*-axis) that share *x* or more with their closest individual. **b** For each value *x* of total shared genome (in cM) on the *X*-axis, we report the median distance (km, computed every 10 cM) for all pairs of (sample, closest individual) who shared at least *x*. Vertical dashed lines indicate the expected value of the total IBD sharing for *k*th degree cousins, computed using 2*G*(1/2)^2(*k*+1)^, where *G* = 7247.14 is the total diploid genome size (in cM) and *k* represents the degree of cousin relationship (e.g. *k* = 2 for second degree cousins, separated by 2(*k* + 1) generations)^11^. The value of 45 observed when no sharing cutoff is considered (i.e. when the *x* value approaches 0) reflects the median prediction error for a random individual, regardless of how much IBD they share with the closest individual.

We hypothesized that the presence of such a fine-scale genetic structure in the UK may be used to effectively predict the birth location of an individual. This would imply that FastSMC may be used to predict other subtle environmental covariates, which may be causing confounding in GWAS^30^. We computed the fraction of individuals who find at least one close genetic relative within the UK Biobank dataset (Fig. 3a). We observed that almost all individuals (99.8%) find a genetic relative with IBD sharing equivalent to a 5th degree cousin (3.5 cM) or closer relationship, with 64.6% of samples finding a putative 3rd degree cousin (56.6 cM) or closer relative. We found that, indeed, stronger genetic ties translate into greater proximity of birth locations (Fig. 3b). For instance, for individuals sharing a fraction of genome equivalent to 3rd degree cousins or closer, the median distance between birth locations is 17 km. Very close genetic relationships are also pervasive in the dataset: about one in four individuals (23.4%) has a relative with genetic sharing equivalent to a 2nd degree cousin (226.5 cM) or closer; for these samples the median distance between birth locations is only 5 km. We then sought to quantify how accurate IBD sharing is at predicting the birth coordinate of a random UK Biobank sample. We found that the birth coordinate of the closest genetic relative for a random UK Biobank individual can be used to predict the individual’s birth location with a median error of 45 km. In contrast, taking a random individual to predict the birth location would result in a median error of 200.6 km (95% bootstrap CI = [200.3, 200.8]). Using this approach to predict a random individual’s current place of living, rather than birth location, resulted in a higher median error of 75 km (Supplementary Fig. 13). Additional details are shown in Supplementary Fig. 14. Prediction in non-White British samples resulted in decreased accuracy due to the smaller sample size (Supplementary Fig. 15B). Conversely, restricting this analysis to only samples of White British ancestry resulted in a slightly improved median error of 42 km (Supplementary Fig. 15A). These findings provide empirical support to recent hypotheses that extensive segment sharing within genealogical databases may be used to recover the genotypes of target individuals^31^, or to re-identify individuals through long-range familial searches^32^.

As expected, entries of the IBD matrix within the past 10 generations are highly correlated to corresponding allele sharing entries of the genetic relatedness matrix (GRM) that is widely used in applications such as principal component analysis^33^ and linear mixed models^34^ (*r* = 0.66, SE = 0.02 computing the GRM for 50,000 random samples using Plink^35^). However, by conditioning on the presence of recent shared ancestors, the IBD matrix is better suited to capture genomic relationships that are recent but would not result in large outlier entries in the GRM. To compare the performance of our IBD-based approach with an approach based on allele sharing in the absence of extremely close relatives, we removed individuals with very recent genetic ties (≤3rd degree relatives, e.g. first degree cousins^2^) and used a simple machine learning approach (K Nearest Neighbors) to perform the prediction of birth coordinates. Prediction of birth coordinates using the IBD matrix was 44% more accurate than prediction based on allele sharing (Supplementary Fig. 16). We also found birth coordinates predicted using IBD sharing to be strongly correlated to true coordinates (*r* = 0.74, 95% CI = [0.73, 0.75], for Y-coordinates and *r* = 0.6, 95% CI = [0.59, 0.62], for X-coordinates), substantially higher than the correlation achieved using allele sharing (*r* = 0.43, 95% CI = [0.41, 0.45], for Y-coordinates and *r* = 0.31, 95% CI = [0.30, 0.33], for X-coordinates).

Analyzing broader patterns of IBD sharing, we found that individuals living in the North of the country share more common ancestry than in the South, and that more generally regions within Scotland, England, and Wales tend to cluster with other regions within the same country. We estimated the effective population size from 300 years ago within each postcode and detected significant correlation (*r* = 0.28, 95% CI = [0.09, 0.47] by bootstrap using postcodes) with present-day population density (Supplementary Fig. 12A). As we look deeper in time, IBD sharing patterns tend to shift and reflect historical migration events within the country. Notably, we find that individuals throughout England share deep genealogical connections with other individuals currently living in the North-West and the North of Wales (Supplementary Fig. 17). These regions correspond to the unromanised regions of the UK and Britons living there are believed to have experienced limited admixture during the Anglo Saxon settlement of Britain occurring at the end of the Roman rule in the 5th century^36^. Elevated IBD sharing between these regions may thus reflect deep genealogical connections within the ancient Briton component of modern day individuals, which is overrepresented in the North-West of the country. As expected, large cosmopolitan regions display substantially more uniform ancestry across the UK. London, in particular, has a uniform distribution of ancestry at deep time scales (Supplementary Fig. 9), suggesting that it has attracted substantial migration for extended periods of time.

### Signals of recent positive selection in the UK Biobank

We analyzed locus-specific patterns of recent shared ancestry, seeking evidence for recent positive selection by identifying loci with unusually high density of recent coalescence times in the UK Biobank dataset. We computed the DRC_50_ (Density of Recent Coalescence) statistic^23^, capturing the density of recent coalescence events along the genome within the past 50 generations (Supplementary Fig. 18). Large values of the DRC_50_ statistic are found at loci where a large number of individuals descend from a small number of recent common ancestors, a pattern that is likely to reflect the rapid increase in frequency of a beneficial allele due to recent positive selection. Although, as expected, the DRC_50_ statistic computed in this analysis is strongly correlated (*r* = 0.67) with the DRC_150_ statistic that was computed using fewer samples from a previous UK Biobank data release by Palamara et al.^23^, the DRC_50_ statistic reflects more recent coalescence events than the DRC_150_ statistic, and thus more specifically reflects natural selection occurring within recent centuries.

**Fig. 4:**
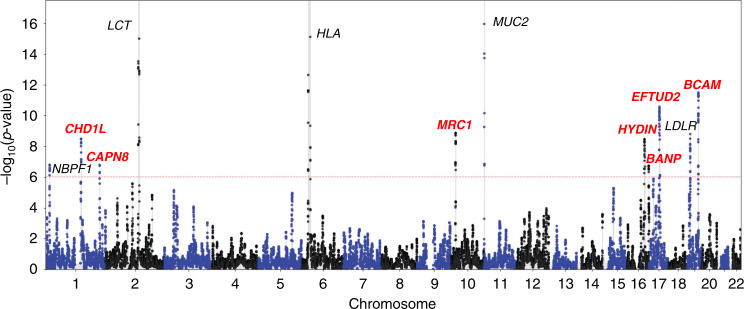
Genome-wide scan for recent positive selection in the UK Biobank dataset. Manhattan plot with candidate gene labels for 12 loci detected at genome-wide significance (adjusting for multiple testing, approximate 1-sided DRC_50 _*p* < 0.05/52,003 = 9.6 × 10^−7^; dashed red line). The DRC_50_ statistic of shared recent ancestry within the past 50 generations was computed using 487,409 samples within the UK Biobank cohort. FastSMC detected 5 loci known to be under recent positive natural selection (gene labels in black) and 7 novel loci (in red). The corresponding *p*-values are reported in Supplementary Table 4.

Analyzing the distribution of the 52,003 windows in the UK Biobank dataset, we detected 12 genome-wide significant loci (at an approximate DRC_50 _*p* < 0.05/52, 003 = 9.6 × 10^−7^; Fig. 4 and Supplementary Table 4). Five of these loci are known to be under recent positive selection, harboring genes involved in immune response (*NBPF1*^37^, *HLA*^38^), nutrition (*LCT*^39^, *LDLR*^40^), and mucus production (*MUC2*^23^). We also identified 7 other loci, harboring genes related to immune response (*MRC1*, playing a role in both the innate and adaptive immune systems^41^, and *BCAM*, encoding the Lutheran antigen system, also associated with low density lipoprotein cholesterol measurement^42^), mucus production (*CAPN8*, involved in gastric mucosal defense^43^), tumor growth (*CHD1L*, associated with tumor progression and chemotherapy resistance in human hepatocellular carcinoma^44^, and *BANP*, encoding a tumor suppressor and cell cycle regulator protein^45^), as well as genetic disorders (*HYDIN*, causing primary ciliary dyskinesia^46^, and *EFTUD2*, causing mandibulofacial dysostosis with microcephaly^47^). We checked that these regions are not extreme in recombination rate or marker density (Supplementary Table 5).

### IBD sharing enables association of ultra-rare variants

**Fig. 5:**
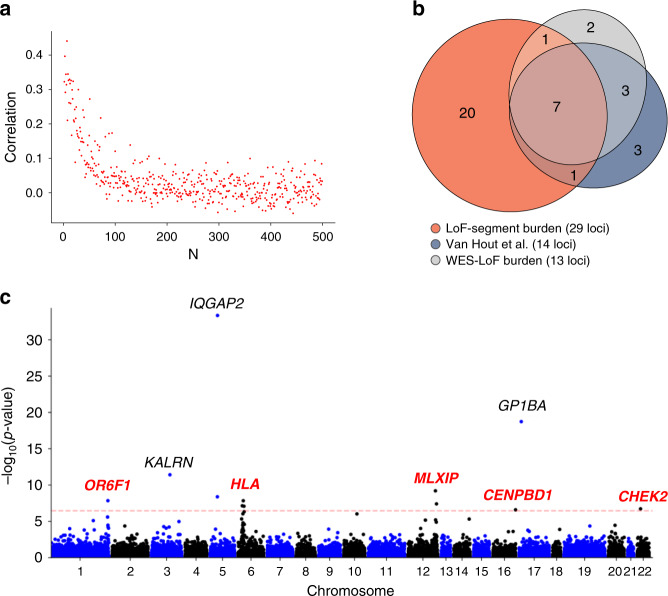
IBD sharing and rare variant associations. **a** Correlation between IBD sharing (average number of IBD segments per pair across UK postcodes in the past 10 generations in the UK Biobank’s 487,409 samples) and ultra-rare variants sharing (average number of *F*_N_ mutations per pair across UK postcodes in the UK Biobank 50k Exome Sequencing Data Release for increasing values of *N*). **b** Venn diagram representing the sets of exome-wide significant associated loci for 7 blood-related traits using three methods: the WES-based LoF burden test reported by Van Hout et al.^49^, a WES-based LoF burden test we performed (WES-LoF burden), and the IBD-based LoF burden test we performed (LoF-segment burden). The corresponding *p*-values were computed using two-sided *t*-tests and are reported in Tables 1, 2 and Supplementary Table 8. **c** Exome-wide Manhattan plot for mean platelet (thrombocyte) volume, after SNP-correction, using 303, 125 unrelated UK Biobank samples not included in the exome sequencing cohort. Labeled genes are exome-wide significant after adjusting for multiple testing: *p* < 0.05/(14,249 × 10) = 3.51 × 10^−7^; dashed red line. Black labels indicate genes that were previously reported by Van Hout et al.^49^ (*KALRN, GP1BA*, and *IQGAP2*), while red labels indicate novel associations detected by our LoF-segment burden analysis. The corresponding *p*-values were computed using two-sided *t*-tests and are reported in Table 2.

Individuals who co-inherit a genomic region IBD from a recent common ancestor are also expected to have identical genomic sequences within that region, with the exception of de-novo mutations and other variants introduced by e.g. non-crossover gene conversion events in the generations leading to their recent common ancestor^48^. We thus expect the sharing of IBD segments to be strongly correlated to the sharing of ultra-rare genomic variants (MAF < 0.0001), which tend to be very recent in origin and are usually co-inherited through recent ancestors who carried such variants. We verified this by testing for correlation between the sharing of IBD segments at various time scales and the sharing of rare variants for the  ~50k individuals included in the UK Biobank’s initial exome sequencing data release^49^ (Supplementary Fig. 19A). Specifically, we analyzed mutations that are carried by *N* out of 99,920 exome-sequenced haploid genomes (for 2 < *N* < 500), which we refer to as *F*_*N*_ mutations^50,51^. We compared the sharing of *F*_*N*_ mutations to the sharing of IBD segments in the past 10 generations within all postcodes in the UK (Fig. 5a). We found that there is indeed a strong correlation between the per-postcode sharing of ultra-rare variants and the per-postcode sharing of recent ancestors (e.g. *r* = 0.3, 95% CI = [0.22,0.37] for *F*_3_ mutations). The correlation between IBD sharing and *F*_*N*_ variant sharing decreases as *N* increases, with slightly higher correlation for more recent IBD segments, while deeper IBD sharing (within 50 generations) tends to provide better tagging of ultra-rare variants of slightly higher frequency (e.g. bootstrap *p* < 0.05/50 = 0.001 for *N* = 20; Supplementary Fig. 19B).

Based on this correlation between sharing of mutations and sharing of IBD segments, we hypothesized that IBD sharing of disease causing mutations would be predictive of disease. In particular, individuals who share an IBD segment with a pathogenic rare variant carrier in a known gene have a higher probability of carrying the pathogenic variant (by inheriting it from the shared ancestor) than the general population, and would thus be at increased risk for the phenotypic effect. The UK Biobank exome pilot^49^ identified multiple rare coding variant burden associations with complex phenotypes, some of which were recently replicated^52,53^. We set out to test whether our IBD inference could empower us to replicate and refine these associations using the larger non-sequenced cohort. For each previously reported gene-phenotype association, we identified all sequenced individuals with a rare LoF variant (mirroring the definition of LoF from Van Hout et al.^49^) and any IBD segments they shared with individuals in the non-sequenced cohort (we refer to these as putative LoF-segments, though they will also include sharing of the non-LoF haplotypes because the phase of the LoF is unknown). As expected due to the effects of natural selection^54^, the LoF variants belonging to a small subset for which an age estimate has recently been computed^55^ are slightly younger than other exome-sequenced variants (Supplementary Table 6). Note that the majority of these variants are singletons or doubletons^49^ and would be excluded from imputation by most current algorithms^56^. Then, in the (independent) non-sequenced cohort, we tested for association between carrying a LoF-segment (the LoF-segment burden) and the phenotype known to be associated with that gene. This approach would be optimal when IBD individuals carry a LoF variant that arose prior to the TMRCA of their shared segment. We thus tested 10 transformations of the LoF-segment metric for association with phenotype to model uncertainty about the age distribution of the underlying causal variants.

Using our LoF-segment burden, we replicated 11 out of 14 previously reported^49^ associations with seven blood-related traits at *p* < 0.05/10 = 0.005 (adjusted for testing of 10 transformations; Table 1). Strikingly, we found eight of these associations to be exome-wide significant in the non-sequenced cohort (*p* < 0.05/(10 × 14,249), reflecting 14,249 genes tested using 10 transformations; Fig. 5b). We next aimed at quantifying how effective IBD sharing is at detecting associations, compared to testing directly based on exome sequencing data. We computed the phenotypic variance explained by the indirect IBD-based test and the direct exome-based test (after subtracting the effect of covariates from both), focusing on the 14 loci reported by Van Hout et al.^49^. The ratio of these variances was 19.64% on average, corresponding to the decrease in effect-size (in units of variance) due to estimation error and inclusion of segments sharing the non-LoF haplotype. We note that, due to phase uncertainty, we expect LoF-segment burden to explain at most 50% of the variance explained by direct sequencing. Assuming the ratio of variances corresponds to the squared correlation between the LoF-segment burden estimate and the true exome burden, the LoF-segment burden estimator has statistical power equivalent to a direct exome sequencing study of 19.64% of the 303, 125, or  ~60k samples^57^ – effectively doubling the size of the exome study. This demonstrates FastSMC’s accuracy and, more broadly, the utility of leveraging distant relatedness in identifying disease associations.

**Table 1:** Comparison between association analyses.

	Gene	Trait	Van Hout et al.	WES LoF burden	LoF-segment burden	${R}_{{\rm{prop}}}^{2}$
1	*IL33*	Eosinophil count	3.30E-10	2.01E-03	8.64E-15	72.26
2	*GP1BA*	Mean platelet (thrombocyte) volume	6.40E-08	8.84E-08	1.82E-19	32.57
3	*TUBB1*	Platelet distribution width	2.50E-23	7.34E-18	7.38E-12	07.25
4	*TUBB1*	Mean platelet (thrombocyte) volume	2.40E-08	3.01E-07	2.15E-03	04.11
5	*TUBB1*	Platelet count	2.10E-09	7.45E-07	4.21E-05	07.84
6	*HBB*	Red blood cell distribution width	5.80E-08	3.49E-02	2.25E-03	23.99
7	*HBB*	Red blood cell count	1.70E-09	7.95E-02	2.68E-02	18.23
8	*KLF1*	Red blood cell distribution width	1.50E-13	6.95E-13	3.49E-34	32.99
9	*KLF1*	Mean corpuscular hemoglobin	1.70E-16	9.11E-15	6.79E-21	16.76
10	*ASXL1*	Platelet distribution width	4.70E-09	1.44E-06	0.16E-00	00.98
11	*ASXL1*	Red blood cell distribution width	2.40E-11	8.23E-04	0.32E-00	01.03
12	*KALRN*	Mean platelet (thrombocyte) volume	2.70E-23	3.85E-18	3.79E-12	07.33
13	*IQGAP2*	Mean platelet (thrombocyte) volume	1.10E-19	3.72E-15	4.40E-34	27.43
14	*GMPR*	Mean corpuscular hemoglobin	1.10E-08	2.94E-06	7.60E-11	22.18

We report association statistics for 14 loci and 7 traits as detected by Van Hout et al.^49^ (obtained using a linear mixed model), our whole-exome sequencing burden analysis (two-sided *t*-test; labeled as WES LoF burden); and the LoF-segment burden (two-sided *t*-test). The Bonferroni-corrected exome-wide significance threshold for the first two approaches is 3.4 × 10^−6^, after correcting for multiple testing with ~15k genes, and 3.51 × 10^−7^ for the LoF-segment burden, after adjusting for 14,249 genes and 10 time transformations. We identify 10 genes at exome-wide significance with the WES-LoF burden test, and we replicate 11/14 at *p* < 0.05/10 = 0.005 (adjusted for testing of 10 transformation) using the LoF-segment association in non-sequenced samples (8 at exome-wide significance). The last column estimates the proportion of the phenotypic variation (${R}_{{\rm{prop}}}^{2}$, in %; Supplementary Table 9) of the sequenced samples that can be explained by the non-sequenced cohort; on average that is 19.64% for all the 14 reported associations, or 27.35% if focusing on the exome-wide significant signals.

Motivated by these results, we next expanded our study to all sequenced genes for this same set of primarily blood-related traits. We identified a total of 186 exome-wide significant gene associations (*p* < 0.05/(10 × 14,249)) spanning 33 genomic loci in the non-sequenced cohort by only leveraging LoF-segments (Table 1); these genes were not significant in the exome burden analysis due to insufficient statistical power. We noticed that some loci included multiple significant associations, suggestive of correlation between associated features as is often seen for GWAS signals. We hypothesized that, in some cases, the true underlying causal variant may be better tagged by known high-frequency SNPs, which are likely to have been detected in previous GWAS analyses. Repeating this analysis including previously associated common variants as covariates reduced the number of significant associations to 111 across 29 loci, suggesting that inclusion of significant common associations in rare variant burden tests may lead to improved interpretability and fewer false-positives due to tagging (Table 2, Fig. 5c and Supplementary Figs. 20–25).

**Table 2:** Associations detected using LoF-segment burden.

	Trait	Chr	Region (Mb)	Min. *p*-value	Candidate gene(s)
1	Eosinophil count	chr6	26.01:31.10	1.21E-26	*HIST1H1A, HIST1H1C, HIST1H1T, HIST1H2BF, HIST1H3E, HIST1H4F, BTN3A2, BTN2A2, BTN3A3, BTN2A1, BTN1A1, ABT1, HIST1H2AG, HIST1H2AH, PRSS16, POM121L2, ZNF391, HIST1H2BM, PGBD1, HIST1H2AK, HIST1H2BO, OR2B2, OR2B6, ZNF165, ZSCAN16, ZKSCAN8, ZSCAN9, ZKSCAN4, NKAPL*, **ZSCAN31**, *ZSCAN12, ZSCAN23, GPX6, PSORS1C2*
2	Eosinophil count	chr9	6.21:6.25	8.64E-15	**IL33**^49,59^
3	Eosinophil count	chr9	135.82:135.86	1.92E-07	**GFI1B**^59^
4	Eosinophil count	chr12	113.01:113.41	7.63E-14	**RPH3A**, OAS3^58^
5	Mean corpuscular hemoglobin	chr6	16.23:16.29	7.60E-11	**GMPR**^49,58,59^
6	Mean corpuscular hemoglobin	chr6	25.72:31.10	3.82E-69	*HIST1H2BA, SLC17A2, HIST1H2AB, HFE*^59^, *HIST1H4C, HIST1H2BD*, **HIST1H4D**, *HIST1H2BG, HIST1H2AE, HIST1H1D, BTN2A1*, *HIST1H2AG, HIST1H4I, ZNF184, PSORS1C2*
7	Mean corpuscular hemoglobin	chr19	12.98:12.99	6.79E-21	**KLF1**^49,58,59^
8	Mean corpuscular hemoglobin	chr22	29.08:29.13	1.43E-07	**CHEK2**
9	Mean platelet thrombocyte volume	chr1	247.87:247.88	1.44E-08	**OR6F1**
10	Mean platelet thrombocyte volume	chr3	123.81:124.44	3.79E-12	**KALRN**^49,59,60^
11	Mean platelet thrombocyte volume	chr5	74.80:76.00	4.40E-34	*POLK*, **IQGAP2**^49,59^
12	Mean platelet thrombocyte volume	chr6	26.18:27.92	1.46E-08	*HIST1H4D*, **POM121L2**, *OR2B6*
13	Mean platelet thrombocyte volume	chr12	122.51:124.49	6.29E-10	**MLXIP**, *ZNF664*
14	Mean platelet thrombocyte volume	chr16	90.03:90.03	2.61E-07	**CENPBD1**
15	Mean platelet thrombocyte volume	chr17	4.83:4.83	1.82E-19	**GP1BA**^49,59^
16	Mean platelet thrombocyte volume	chr22	29.08:29.13	1.93E-07	**CHEK2**
17	Platelet count	chr1	43.80:43.82	1.99E-07	**MPL**
18	Platelet count	chr5	75.69:76.00	6.52E-08	**IQGAP2**^59^
19	Platelet count	chr6	26.59:26.60	1.1E-07	**ABT1** (within *HLA* region)
20	Platelet count	chr12	109.88:113.33	4.82E-13	*KCTD10*, *TCHP*, **RPH3A**^60^
21	Platelet count	chr17	4.83:4.83	1.43E-07	**GP1BA**^59,60^
22	Platelet distr. width	chr11	116.66:116.66	1.94E-08	**APOA5**
23	Platelet distr. width	chr17	4.83:4.83	4.26E-09	**GP1BA**^59^
24	Platelet distr. width	chr20	57.59:57.60	7.38E-12	**TUBB1**^49,59^
25	Red blood cell count	chr6	26.45:31.10	1.39E-10	*BTN2A1, POM121L2, HIST1H2BM, HIST1H2BO, ZNF165*, **ZSCAN9**, *ZKSCAN4, PGBD1, ZSCAN31, GPX6, PSORS1C2*
26	Red blood cell distr. width	chr6	26.01:28.48	3.03E-15	*HIST1H1A, HIST1H3A, HIST1H1C, HIST1H1T, HIST1H4D, HIST1H4F, BTN3A2, HIST1H2AG, HIST1H2AH*, **ZNF391**, *HIST1H2BM, HIST1H2AM, ZNF165, ZSCAN16, NKAPL, PGBD1, ZSCAN31, ZSCAN12, GPX6*
27	Red blood cell distr. width	chr9	135.82:135.86	8.1E-08	**GFI1B**^58,59^
28	Red blood cell distr. width	chr11	116.69:116.70	3.67E-11	**APOC3**
29	Red blood cell distr. width	chr19	12.98:12.99	3.49E-34	**KLF1**^49^

Exome-wide significant associations (after adjusting for multiple testing *p* < 0.05/(14,249 × 10) = 3.51 × 10^−7^) detected using LoF-segment burden (SNP-adjusted). Associated genes are clustered in 29 loci. For each locus we report the set of associated genes and minimum *p*-value. The gene corresponding to the minimum *p*-value is highlighted in bold.

Our exome-wide significant signals include the association between platelet count and *MPL* (two-sided *t*-test *p* = 1.99 × 10^−7^), which encodes the thrombopoietin receptor that acts as a primary regulator of megakaryopoiesis and platelet production and has not been previously implicated by genome-wide scans of either rare or common variants. We also detect several associations in genes that were not detected using exome sequencing but have been previously implicated in genome-wide scans for common variants, including associations between eosinophil count, *GFI1B* (two-sided *t*-test *p* = 1.92 × 10^−7^) and *RPH3A* (two-sided *t*-test *p* = 7.63 × 10^−14^)^58,59^, and associations between platelet count, *IQGAP2* (two-sided *t*-test *p* = 6.52 × 10^−8^) and *GP1BA* (two-sided *t*-test *p* = 1.43 × 10^−7^)^59,60^. We also identify genes that were previously associated with other blood-related phenotypes in other populations, including the association between platelet distribution width and *APOA5* (two-sided *t*-test *p* = 1.94 × 10^−8^), a gene that encodes proteins regulating the plasma triglyceride levels; common variants linked to this gene have been linked to platelet count in individuals of Japanese descent^61^. We detect association between red blood cell distribution width and *APOC3* (two-sided *t*-test *p* = 3.67 × 10^−11^), a gene encoding a protein that interacts with proteins encoded by other genes (*APOA1, APOA4*) associated with the same trait. The association between *APOC3* and platelet count was also detected with our WES-LoF burden analysis (two-sided *t*-test *p* = 2.13 × 10^−7^) and by previous studies based on common SNPs^59^. We also found an association between *CHEK2* and both mean corpuscular hemoglobin (two-sided *t*-test *p* = 1.43 × 10^−7^) and mean platelet volume (two-sided *t*-test *p* = 1.93 × 10^−7^). This gene plays an important role in tumor suppression and was found to be associated with other blood traits, such as platelet crit, using both exome sequencing^49^ and common GWAS SNPs^59^, and red blood cell distribution width^58^. Overall, this analysis highlights the utility of applying FastSMC on a hybrid sequenced/genotyped cohort to identify novel, rare variant associations and/or characterize known signals in larger cohorts.

## Discussion

We developed FastSMC, an algorithm for IBD detection that scales well in analyses of very large biobank datasets, is more accurate than existing methods, enables estimating the time to most recent common ancestor for IBD individuals, and provides an estimate of uncertainty for detected IBD regions. We leveraged FastSMC to analyze 487,409 British samples from the UK Biobank dataset, detecting  ~214 billion IBD segments transmitted by shared common ancestors in the past  ~1500 years. This enabled us to obtain high-resolution insight into recent population structure and natural selection in British genomes. Lastly, we used IBD sharing between exome sequenced and non-sequenced samples to infer the presence of LoF variants, successfully replicating known burden associations with seven blood-related complex traits and revealing novel gene-based associations.

The level of geographic granularity that could be captured by the IBD networks emerging from our analysis underscores the importance of modeling distant relationships in genetic studies. Indeed, detecting IBD segments among close and distant relatives is a key step in many analyses, such as genotype imputation or haplotype phase inference^5,14,15^, haplotype-based associations^12,13^, as well as in the estimation of evolutionary parameters such as recombination rates^62,63^, or mutation and gene conversion rates^48,64^. More broadly, the observed geographic heterogeneity in IBD sharing and fine-scale structure is a reminder that human populations substantially deviate from random mating, even within small geographic regions. In particular, efforts to generate optimal sequenced reference panels for imputation^65^ may be greatly improved by directly sampling based on distant relatedness. Our findings also provide empirical support to recent hypotheses that relatedness within available genomic databases is sufficiently pervasive to enable recovering the genotypes of target individuals^31^, or to re-identify individuals through familial searches^32^. As demonstrated by our analysis, leveraging IBD to impute ultra-rare variant burden from small sequenced reference panels can be an effective approach to reveal gene associations in complex traits and diseases. Although our analysis only considered association to individual genes, in principle genome-wide IBD sharing could also be leveraged to enhance common SNP-based risk prediction, which is particularly relevant for non-European cohorts where sequencing is limited.

FastSMC inherits some of the limitations and caveats of the GERMLINE and ASMC algorithms. First, as with most IBD detection methods, FastSMC requires the availability of phased data. We note, however, that when very large cohorts are analyzed, long-range computational phasing results in high-quality haplotype estimation with switch errors rates as low as one every several tens of centimorgans^5,14^. This is particularly true in regions that harbor IBD segments, which are of interest in our analysis. Nevertheless, our analysis has shown the presence of substantial regional heterogeneity in the extent to which individual genomes are spanned by IBD segments, which is likely reflected in a heterogeneous quality of phasing, as well as other downstream analyses, such as imputation, that rely on the presence of IBD segments. Second, FastSMC requires the input of a demographic model and allele frequencies as a prior in order to accurately estimate the age of IBD segments, and is thus subject to biases whenever the demographic model is misspecified. Although we have verified that these biases are not substantial, future work may enable us to simultaneously estimate IBD sharing and demographic history. Like ASMC, FastSMC may tolerate reasonable levels of model misspecification, but a user should be aware that issues such as substantial inaccuracies in the genetic map or strong heterogeneity of genotyping density or quality may lead to biases. Third, FastSMC currently does not enable analysis of imputed data, a limitation that is shared by other IBD detection methods. Finally, the accurate identification of extremely short IBD segments (<1 cM) spanning hundreds of generations remains a challenge, both computationally (as the number of such segments increases very rapidly) and methodologically (as fewer variants are available to provide signal for distinguishing IBS from IBD). This underscores the importance of quantifying uncertainty through estimates such as IBD segment quality, precision, and recall, which are affected by parameter choices for FastSMC and other IBD detection methods. Our analysis detected an average of 1.8 IBD segments per pair in the UK Biobank dataset within the past 50 generations. This is consistent with a previous study focusing on longer and more recent segments (average of 0.1 segments  >2.9 cM per pair^66^), but less than another recent study in a similar length range (average 1.96 segments  >2 cM per pair^67^). Taking uncertainty of the detected IBD segments into account may reconcile these estimates.

In addition to algorithmic improvements to address the limitations above, we believe there are a number of interesting future extensions and interactions with other existing methods in this area. FastSMC’s identification step currently relies on the GERMLINE2 genotype hashing strategy. It will be interesting to test other heuristic strategies for rapidly identifying identical segments, such as the locality-sensitive hashing strategy recently implemented in the iLASH algorithm (exhibiting 95% concordance with GERMLINE in application to real multi-ethnic data^66^), or methods that rely on the positional Burrows-Wheeler transform (PBWT) data structure^17,67,68^. Several methods now exist to reconstruct gene genealogies in large samples^69–72^. Two recent methods substantially improved the scalability of this type of analysis, but they either focus on data compression, relying on fast heuristics to achieve scalability at the cost of deteriorating accuracy in sparse array data^71^, or employ further modeling that requires sequencing data^72^, with a computational cost that is quadratic in sample size (the same computational complexity required to run the full ASMC algorithm on all sample pairs). It is of continued interest to identify synergies between fast heuristic and accurate probabilistic approaches, which can lead to computationally efficient methods that remain robust to real world data heterogeneity. Although in this work we focused on large modern biobanks comprising SNP array data, sequencing datasets are quickly becoming available. FastSMC may be tuned to enable the analysis of sequencing data as well. Finally, looking at downstream applications, a direction of future work will be to leverage FastSMC to better control for subtle population stratification for both rare and common variants in association studies. Our results show that geographic coordinates can be effectively inferred from recent IBD sharing, and suggest that this may be a path towards capturing subtle environmental covariates^30^ that are missed by genome-wide IBS-based approaches. FastSMC’s output could thus account for subtle stratification even when the non-genetic confounder has a small and sharp distribution, where methods such as genomic control, PCA, or mixed models have limited efficacy^73^.

## Methods

### FastSMC identification step

FastSMC’s identification step leverages genotype hashing, a strategy that was introduced by the GERMLINE algorithm^19^ to obtain substantial gains in computational scalability in the detection of pairwise shared IBD segments. This approach restricts the search space of IBD pairs to those that have small, identical shared segments, which are then extended to long segments with some tolerance. This in turn reduces the IBD search from all pairs of individuals to the subset of pairs that produce a hash collision at a given segment plus the cost of hashing the genotype data, which is linear in sample size and genome length, thus dramatically reducing the cost of IBD detection. However, a limitation of this strategy is that certain short haplotypes can be extremely common in the population and result in hash collisions across a large fraction of samples, effectively reverting back to a nearly all-pairs analysis and monopolizing computation time (Supplementary Fig. 1, gray). These common haplotypes are likely due to recombination cold-spots and typically contain little variation to classify shared segments. The majority of computation is thus spent processing regions with the least information content. The GERMLINE2 algorithm, which we developed in this work, proposes an adaptive hashing approach that adjusts to local haplotype complexity to dramatically reduce computational and memory requirements. GERMLINE2 proceeds as follows: (1) the input haplotype data is divided into small windows containing 16 or 32 SNPs each (depending on memory architecture); for a given window *w*, (2) all haplotypes are converted to binary sequences and efficiently hashed into bins of identical segments; (3) for each bin that contains more individuals than a fixed threshold (i.e. a low complexity bin) step 2 is recursively performed for window *w* + 1 until no more low complexity bins are found; (4) all pairs of individuals sharing within a bin are then recorded in a separate hash table that stores putative segments; (5) pairs of individuals sharing contiguous windows that are sufficiently long are reported for validation. The primary computation speed-up comes from the recursive hashing step, which requires haplotypes to be sufficiently diverse before they are explored for pairwise analysis and stored (Supplementary Fig. 1, green). To allow for possible phasing errors, a putative shared segment is maintained through a parameterized number of non-identical windows, and the total number of non-identical windows within the segment is also reported for filtering. Most phasing errors either appear as blips, where a phase switch is immediately followed by a switch back, or by single switches followed by long stretches of accurate phase^14,15^ – both of which are permitted by allowing periodic non-matching along the putative IBD segment. This permissive treatment of phasing is further filtered in the validation step (below). GERMLINE2 thus does not require any backtracking and only a small number of physical windows need to be stored in memory at any time (only enough to perform the recursion), allowing the method to run on input data of unlimited length.

### FastSMC validation step

Every segment detected by GERMLINE2 in the identification step is added to a buffer of candidate segments. These segments are immediately decoded by the ascertained sequentially Markovian coalescent (ASMC) algorithm^23^ once the buffer is full. ASMC is a coalescent-based HMM^24,25,27,74^ that estimates the posterior of the coalescence time, or TMRCA, for a pair of individuals at each site along the genome using either sequencing or SNP array platforms. It leverages a demographic model as prior on the TMRCA, which increases accuracy in detecting regions of low TMRCA^26^, but would be infeasible to apply to the analysis of all pairs of genomes, and is thus only applied with the goal of validating previously identified candidate IBD regions. The hidden states of the HMM are discretized intervals, corresponding to a user-specified set of TMRCA intervals. The HMM emissions probabilities correspond to the probabilities of observing both the genotypes of the pair of analyzed individuals and the frequencies of mutations along the sequence, given the pair’s TMRCA at each site, and the frequency of the allele^27^. The HMM transitions between hidden states correspond to changes in TMRCA along the genome due to recombination events, based on the conditional Simonsen-Churchill model^75,76^. The demographic history of the analyzed haplotypes is first estimated using other methods^25,27^ and provided in input, so that the initial state distribution, the transition, and the emission probabilities can be computed. The most likely posterior sequence of TMRCAs along the genome is inferred using a dynamic programming approach that requires computing time linear in the number of hidden states, leading to a substantial speed-up over an HMM’s standard forward-backward algorithm, which scales quadratically in the number of hidden states. When decoding the buffer of candidate segments, ASMC computes the posterior of the coalescence time for each candidate segment and each site from the minimum starting position to the maximum ending position in the buffer. At each site, if the posterior of coalescence time being between present time and the user-specified time threshold is higher than its prior, the site is considered to be IBD and the IBD segment is extended to the next site if the same condition is still satisfied, obtaining multiple IBD segments, all shorter than the original IBD candidate segments. The average probability of the TMRCA being between present time and the user-specified time threshold is computed over all sites until the segment breaks. This average probability corresponds to an IBD quality score: the higher it is, the more likely the segment is IBD. Each segment is also associated with an age estimate corresponding to the average MAP along the segment. FastSMC finally outputs each IBD segment with its corresponding IBD quality score and age estimate.

### FastSMC parallelization

We divided the genome in 39 autosomal regions from different chromosomes or separated by centromeres^23^. This enabled us to efficiently parallelize analyses on the UK Biobank dataset, and prevented issues due to low marker density in centromeres. In addition, FastSMC enables a user to parallelize the analysis by specifying the total number of computing jobs to be run in parallel. If the user requests to run *K* independent parallel jobs, the total number of $$\left(\begin{array}{c}N \\ 2\end{array}\right)$$ sample pairs to be analyzed are subdivided into *K* groups of approximately equal number of pairs. More in detail, the samples are divided into $S=\frac{1+\sqrt{1+8K}}{2}$ disjoint sets of approximately the same size, where *K* is such that *S* is an integer. Each parallel job then processes a unique pair of sets {*i*, *j*} for 1 ≤ *i* ≤ *j* and 1 ≤ *j* ≤ *S*. Note that only samples in sets *i* and *j* are loaded in memory, which leads to substantially lower memory footprint when large data sets are processed. IBD detection is then only performed for the *N*_*i*_ × *N*_*j*_ pairs across sets *i* and *j* if *i* ≠ *j*, or for the $$\left(\begin{array}{c}{N}_{i}\\ {2}\end{array}\right)$$ pairs within set *i* if *i* = *j*. We leveraged this approach to extensively parallelize the analysis of the UK Biobank dataset.

### Simulations

Unless otherwise specified, all simulations use the setup of Palamara et al.^23^, which is described in this section. We used the ARGON simulator (v.0.1.160615)^77^, incorporating recombination rates from a human chromosome 2 and a recent demographic model for European individuals (Northern European [CEU] population^27^). For each dataset, we simulated 300 haploid individuals and a region of 30 Mb. To simulate SNP array data, we subsampled polymorphic variants to match the genotype density and allele frequency spectrum observed in the UK Biobank dataset. We used recombination rates from the first 30 Mb of chromosome 2 (average rate of 1.66 cM per Mb). No genotyping or phasing error was introduced in our simulations. We simulated one dataset following this setup to fine-tune parameters, and 10 other datasets (all with different seeds) for accuracy benchmarking. The demographic model and genetic map used to simulate the data were used when running FastSMC, unless otherwise specified. When testing FastSMC’s robustness to demographic model misspecification, we simulated data under a constant population size of 10,000 diploid individuals, but ran FastSMC assuming a European demographic model.

### Accuracy evaluation

We compared FastSMC to the most recent published software version available for existing methods at the time we conducted this analysis: germline-1-5-2, refined-ibd.23Apr18.249 and RaPID_v.1.2. Throughout this work, we define a genomic site to be shared IBD by a pair of phased haploid individuals if their TMRCA at the site is lower than a specified time threshold (e.g. 50 generations). This is a natural definition for IBD sharing, as it is closely related to several other quantities that are of interest in downstream analyses, such as genealogical relatedness or the probability of sharing rare genomic variants. We note, however, that a number of other definitions can be found in the literature^22^. This is often due to the fact that current IBD detection algorithms cannot effectively estimate the TMRCA of a putative IBD segment. Downstream analyses of shared segments (e.g. refs. ^6,9^) thus often resort to using the length of detected segments as a proxy for its age, since a segment’s length is expected to be inversely proportional to its TMRCA. We benchmarked all methods using several such time thresholds (25, 50, 100, 150, and 200 generations), testing all polymorphic sites for all pairs of genomes in the simulated data, across 10 coalescent simulations. Accuracy was quantified using the area under the precision-recall curve (auPRC), which effectively addresses issues with class imbalance that are expected in this analysis due to the low prevalence of IBD sites compared to non-IBD sites. Precision represents the fraction of identified sites that are indeed IBD (following the TMRCA-based definition of IBD), and recall represents the fraction of true IBD sites that are successfully identified. Particularly, for a given IBD time threshold, a site inferred to be IBD by one of the methods was considered correct (true positive) if the true TRMCA at this site was indeed below the specified IBD time threshold, and incorrect (false positive) if the true TRMCA at this site was above the IBD time threshold. Similarly, a site that was not reported to be IBD by the tested method was considered correct/incorrect (true/false negative) if the true TMRCA at the site was found to be below/above the IBD time threshold. We used these definitions to compute the precision and recall values for all methods. Each method presents different parameters, which can be used to tune precision and recall, e.g. by allowing a more or less permissive detection of IBD segments. String-matching methods (GERMLINE and RaPID) report long, approximately IBS regions and do not produce calibrated estimates of segment quality. A commonly used proxy for the likelihood of a detected IBS segment being IBD is its length, with longer IBS segments being more likely to be IBD than shorter ones. In lack of an interpretable measure of accuracy, precision and recall for the output of GERMLINE and RaPID was thus tuned by using different segment length cutoffs. RefinedIBD and FastSMC, on the other hand, both provide an explicit quantification of segment quality. RefinedIBD outputs a LOD score for each segment, while FastSMC computes a segment’s IBD quality score, which is the posterior of the TMRCA being between present time and the user-specified time threshold. We thus used LOD score and IBD quality score to tune precision and recall of RefinedIBD and FastSMC, respectively. Each method presents a number of additional parameters, which we further optimized using a grid-search, so that each method can be run with a set of parameters that is as close to optimal as possible. Despite the extensive tuning, not all accuracy values could be explored by all methods. Namely, some recall values cannot be achieved using realistic parameters, due to factors such as the minimum allowed LOD parameter for RefinedIBD, the time discretisation introduced in FastSMC and the minimum length parameter for all methods. We thus evaluated all algorithms by restricting the comparison to the range of recall values that could be achieved by all methods, which we refer to as common recall range. Furthermore, some of these parameters affect the speed of each algorithm. The parameters we chose for comparing methods are optimized for maximum accuracy, although we avoided parameters that would result in degeneracies (e.g. the minimum length in FastSMC’s identification step could be set to values below 0.1 cM, effectively disabling this step and reverting to a pairwise ASMC analysis, which would lead to a higher accuracy and larger recall range, at the cost of unreasonable computation).

### Fine-tuning of methods

For each method (FastSMC, GERMLINE^19^, RefinedIBD^20^, and RaPID^21^) and each IBD time threshold (25, 50, 100, 150, and 200 generations), we performed a grid-search over possible parameter values to optimize the accuracy on one simulated dataset and select the best set of parameters. For each method, we then explored the obtained set of parameters to make the algorithm faster while negligibly compromising the accuracy (in most cases, this resulted in a slightly smaller recall range but with a substantial gain in speed). We finally used 10 independent simulated datasets to validate the accuracy and the UK Biobank dataset to measure running time and memory usage (Supplementary Figs. 4–5). Unless otherwise specified, the parameters presented in Supplementary Table 1 were used in all analysis.

### Computing confidence intervals

Unless otherwise indicated, confidence intervals (CIs) were computed by bootstrap using 39 genomic regions as resampling unit. The use of genomic regions as resampling unit, rather than individuals, ensures that approximately independent bootstrap replicates are utilized. These 39 genomic regions were obtained by dividing the genome (autosomal chromosomes only) in regions from different chromosomes or separated by centromeres.

### Estimating the age of an IBD segment

A common way to estimate the age of an IBD segment is to use its length to obtain a maximum likelihood estimator (MLE). When the time in generations *g* to the most recent common ancestor is known, the total length of a randomly chosen shared IBD segment follows an exponential distribution with rate 1/2*g* per Morgan^6^. The likelihood function is thus given by ${\mathcal{L}}(g)=(2g){e}^{-2\mathrm{lg}\,}$, where *l* denotes the segments length in Morgans. As $\frac{{\rm{d}}{\mathcal{L}}}{{\rm{d}}g}(g)=2{e}^{-2\mathrm{lg}\,}(1-2\mathrm{lg}\,)$, the MLE is given by $\hat{g}=\frac{1}{2l}$. FastSMC does additional modeling and provides a different age estimate, which consists in the average MAP estimate of the TMRCA along the segment. We sometimes report segment age estimates in years, rather than generations, assuming 30 years per generation.

### Effective population size estimate

Assuming a constant effective size *N*_e_, the probability of finding a common ancestor at a given site for a pair of individuals is exponentially distributed with mean *N*_e_. The probability of finding a common ancestor before any time threshold *T* is thus given by ${\rm{P}}({t}_{{\rm{TMRCA}}}\le T| {N}_{{\rm{e}}})=\mathop{\int}\nolimits_{0}^{T}\frac{1}{{N}_{{\rm{e}}}}{e}^{\frac{-t}{{N}_{{\rm{e}}}}}\mathrm{dt}\,=1-{e}^{\frac{-T}{{N}_{{\mathrm{e}}}}}\simeq \frac{T}{{N}_{{\mathrm{e}}}}$ for *N*_e_ → *∞* i.e ${N}_{{\rm{e}}}=\frac{T}{{\rm{P}}({t}_{{\rm{TMRCA}}}\le T| {N}_{{\rm{e}}})}$ for *T* > 0. We estimate this probability using the fraction of genome shared by IBD segments denoted by *f*_*T*_. Let *Γ* denote the set of sites along the genome and *θ* the demographic model. ${\mathbb{E}}({f}_{T}| \theta )={\mathbb{E}}\left(\frac{1}{| \Gamma | }{\sum }_{\gamma \in \Gamma }{1}_{\theta }({t}_{{\rm{TMRCA}}}<T\,\,{\text{at}}\,\, {\text{site}}\,\,\gamma )\right)=\frac{1}{| \Gamma | }{\sum }_{\gamma \in \Gamma }{\mathbb{E}}\left({1}_{\theta }({t}_{{\rm{TMRCA}}}<T\,\,{\text{at}}\,\, {\text{site}}\,\,\gamma )\right)=\frac{1}{| \Gamma | }{\sum }_{\gamma \in \Gamma }{\rm{P}}({t}_{{\rm{TMRCA}}}\le T\,\,{\text{at}}\,\, {\text{site}}\,\,\gamma | \theta )={\rm{P}}({t}_{{\rm{TMRCA}}}\le T| \theta )$. We finally estimate the effective population size *N*_e_ using $\hat{{N}_{{\rm{e}}}}=\frac{T}{\overline{{f}_{T}}}$ for any *T* > 0, where $\overline{{f}_{T}}$ is the sample mean for the fraction of genome shared *f*_*T*_. Note that, for simplicity, we infer a single aggregate effective population size across the past *T* generations rather than comparing more complex demographic models.

### UK Biobank dataset and definition of postcodes

The UK Biobank cohort^2^ contains 487,409 samples, which were phased at a total of 678,956 autosomal biallelic SNPs using Eagle2^15^. In all, 49,960 of these individuals were also exome-sequenced resulting in  ~4 million polymorphic variants, 98.4% of which have frequency <1%^49^. We used the same sets of unrelated individuals (*N* = 407,219) and individuals of self-reported and inferred White British ancestry (*N* = 408,974) as defined by Bycroft et al.^2^. Related individuals refer here to ≤3rd degree relatives, e.g. first degree cousins, estimated using the software KING^78^. The UK Biobank cohort predominantly contains samples of White British ancestry^2^ and the inclusion of non-White British samples did not result in substantial biases for analyses of population structure and natural selection. We thus decided not to exclude samples based on ancestry, unless otherwise specified, so that results are representative of the average UK Biobank participant. FastSMC was run on the UK Biobank data set using the optimal set of parameters for IBD detection within the past 50 generations, using the demographic model for CEU individuals inferred by Terhorst et al.^27^. We note that several candidate demographic models could be adopted^9^ and that simultaneous estimation of IBD sharing and demographic model are an attractive direction of future investigation.

Birth coordinates (all within the UK) were available for 432,968 individuals in the cohort in the Ordnance Survey Great Britain 1936 (OSGB36) Eastings and Northings system. We refer to these coordinates as X and Y coordinates, which can be converted into longitude and latitude. Home addresses at assessment were available for 482,832 samples in the OSGB36 Eastings and Northings system. We refer to these coordinates as home addresses. We analyzed population structure using 120 postcodes in the UK, only looking at the first one or two letters indicating the city or region. Postcodes BT, BF, BN, and CR were not included due to lack of samples.

### Hierarchical clustering

We constructed a similarity matrix for all individuals with birth coordinates in the UK Biobank dataset (432,968 samples) using the sharing of IBD segments within the past 10 generations. This resulted in a sparse 432,968 × 432,968 matrix, where entry (*i*, *j*) corresponds to the fraction of genome shared by common ancestry in the past 10 generations between individuals *i* and *j*. We computed the largest connected component of this matrix, which comprised all but 102 individuals, which we excluded from further analysis. We then applied an Agglomerative Hierarchical Clustering algorithm for Sparse Similarity Matrices using average linkage with the sparseAHC library. We obtained a dendrogram with 432,866 leaves (one for each sample) and a single root. Each node is annotated with a distance from the root, ranging from 0 (the root itself) to 1 (the leaves). A node’s distance from the root corresponds to the fraction of genome shared by individuals whose TMRCA is such a node. To visualize clustering of individuals in Fig. 2, we cut the tree at increasingly large distances from the root, corresponding to increasingly fine-grained clusters in terms of both genetic and geographic proximity of the samples. In each case, we only highlight large clusters containing at least 500 individuals. To plot results, we divided each submap into 10,080 grid cells (80 lines along the *X*-axis and 126 lines along the *Y*-axis). In each cell, we computed the most represented cluster (i.e the cluster with the largest number of individuals in that cell) and individuals from that cluster are shown in the corresponding color. The transparency of all points within a cell (ranging from 0 to 1) was set to the fraction of individuals from that cell corresponding to the most represented cluster. All light gray dots correspond to individuals that are either in clusters containing <500 samples or part of a cluster different from the one represented in the grid cell. Including/excluding individuals of non-White British ancestry has a negligible impact on this analysis, as most of these samples belong to smaller clusters not represented in Fig.  2 (in light gray).

### K-NN prediction of birth location

We randomly sampled two subsets of 10,000 individuals each from the UK Biobank cohort and used the sharing of recent common ancestors in the past 600 years with the remaining 412,866 individuals in the UK Biobank dataset to predict their birth locations, applying the K Nearest Neighbors algorithm. One dataset was used to find the optimal value for the parameter *K*, while the second one was used to validate the results (details shown in Supplementary Fig. 16). We computed pairwise genetic similarity across individuals using either FastSMC-estimated pairwise IBD sharing, or using a standard estimate of kinship based on genome-wide allele sharing. This kinship estimator was obtained by computing the product **XX**^⊤^, where **X** is the *N* × *S* genotype matrix (*N* = 432,866 samples and *S* = 716,175 autosomal SNPs), standardized to have mean 0 and variance 1 for each column. Sharing of very close relatives is highly informative of geographic proximity. We thus excluded ≤3rd degree relatives^2^ from the dataset to bypass this source of information and test the generality of this approach. Prior to the exclusion of close relatives, the IBD-based predictor obtained an average error of 86 km (95% CI = [83,88], optimal *K* = 1), while the allele sharing distance predictor obtained an average mean error of 118 km (95% CI = [115,121], optimal *K* = 1). After removing close relatives (which brought sample size down to 8226), the IBD-based predictor obtained an average error of 95 km, 95% CI = [93,97], optimal *K* = 5 compared to 137 km, 95% CI = [135,139], optimal *K* = 5 for allele sharing. Note that this analysis focused on mean error, which is larger than median error (e.g. as in Fig. 3) due to the presence of outliers.

We regressed the true *X* (resp. *Y*) birth coordinates on the predicted *X* (resp. *Y*) birth coordinates using either IBD sharing in the past 20 generations allele sharing, for the set of 8226 random samples we obtained after excluding 3rd degree relatives^2^. The estimated coefficient for the IBD-based predictor was 0.91, 95% CI = [0.88, 0.94], (resp. 0.96, 95% CI = [0.94, 0.98]), substantially larger than the estimated coefficient for the allele sharing predictor (0.12, 95% CI = [0.09,0.16]; resp. 0.12, 95% CI = [0.09, 0.15]). Finally, after excluding close relatives, we computed the correlation between true and predicted coordinates for both methods, obtaining a stronger correlation when using IBD sharing (*r* = 0.6 for *X* coordinate and *r* = 0.74 for *Y* coordinate) than when using allele sharing (*r* = 0.31 for *X* coordinate and *r* = 0.43 for *Y* coordinate, respectively). Correlation for IBD sharing was also stronger without removing close relatives (*r* = 0.63 for *X* coordinate and *r* = 0.77 for *Y* coordinate, compared to *r* = 0.40 and *r* = 0.42 respectively for allele sharing). We did not exclude individuals from these analyses based on their inferred or reported ancestry, so that these results are reflective of the average UK Biobank participant. Prediction accuracy for non-White British individuals will be lower due to a smaller sample size. We thus expect that restricting these analyses to White British samples will lead to slightly improved accuracy.

### Detection of recent positive selection

In order to identify genomic regions with an usually high density of coalescence times, we computed the Density of Recent Coalescence (DRC_*T*_) statistic within the past *T* generations^23^. FastSMC does not output the posterior of the TMRCA but provides an IBD quality score, corresponding to the sum of posterior probabilities between generations 0 and *T*, where *T* is the user-specified threshold. As the UK Biobank dataset was analyzed for *T* = 50, the DRC_50_ statistic at a given site along the genome was estimated by averaging all IBD quality scores obtained from all analyzed pairs of samples (assuming a score of 0 if no segment is present for a pair). The DRC_50_ statistic reflects the probability that a random pair of individuals coalesced at a given genomic site during the past 50 generations. We averaged it within windows of 0.05 cM along the genome. Results are presented in Fig. 4 and Supplementary Table 4.

Given *n* samples from a population of recent effective size *N*, the DRC_50_ statistic is approximately Gamma-distributed under the null for *n* ≪ *N*^23^. We then built an empirical null model using the database of regions under positive selection used by Palamara et al.^23^. We fitted a Gamma distribution (using the Scipy library^79^) to the estimated DRC_50_ values within putative neutral regions (after excluding the regions of known positive selection and 500 kb windows around them), and used this model to obtain approximate one-sided *p*-values throughout the genome. We analyzed 52,003 windows, using a Bonferroni significance threshold of 0.05/52,003 = 9.6 × 10^−7^. When multiple candidate genes were found, we only retained the one nearest to the top SNP (i.e with smallest *p*-value). Three of the genome-wide significant signals we detected (*MRC1* locus, chr10:17.43-18.10 Mb; *HYDIN* locus, chr16:70.10-72.69 Mb, and *EFTUD2* locus, chr17:41.84-44.95 Mb) fell within the putative neutral regions of the genome. We thus iterated this procedure, excluding these loci from the set of putative neutral loci. Once again, one of the genome-wide significant loci (*BANP* locus, chr16:88.25-88.48 Mb) overlapped with the putative neutral regions. We excluded this locus and iterated the procedure again. Results from the empirical null model fitting are presented in Supplementary Fig. 18. Finally, we verified that the genome-wide significant peaks detected using this approach are not found in regions of extremely high recombination rate or low marker density, which may introduce systematic biases in IBD detection Supplementary Table 5. This analysis was replicated after excluding samples of non-White British ancestry but resulted in the same set of genome-wide significant loci.

We note that computing significance for the DRC_50_ statistic using this approach relies on several simplifying assumptions. Specifically, we assume that all pairs of individuals coalesce independently within the past 50 generations, which is not conservative and may be violated in practice. On the other hand, we fit the null model using real data, which likely contains regions under weak selection, and neglect the correlation across DRC estimates in different bins, applying a Bonferroni correction; both are conservative choices. Overall, using this approach to assess significance for the DRC statistic within small time windows was observed to not lead to significant inflation^23^ (Supplementary Fig. 18), although the resulting approximate *p*-values should be interpreted with care.

### Association analyses

We used each IBD segment between exome-sequenced LoF carrying individuals and non-sequenced individuals as a surrogate for the latter carrying an untyped LoF mutation, which we then tested for association with phenotype. For a given gene and a given non-sequenced individual, we define a LoF-segment as any IBD segment shared with an exome-sequenced LoF mutation carrier. We then compute a LoF-segment burden for each individual as the sum of probabilities (IBD quality scores) of all LoF-segments involving that individual, under the assumption that increased IBD probability and incidence corresponds to increased probability of sharing the LoF variant. Finally, this burden is tested for association with each target phenotype (rank-based inverse normal transformed) in a linear regression with covariates for age, sex, BMI, smoking status, and four principal components (two-sided test), similarly to the study from Van Hout et al.^49^.

Although this test captures uncertainty about the sharing of IBD segments through the use of IBD quality scores, it makes use of all LoF-segments, regardless of their age. As a result, it may be suboptimal in cases where the LoF arose after the TMRCA, for which a LoF-segment is independent of underlying LoF sharing, and thus do not contribute signal to the burden test. We thus augmented the LoF burden test by separately considering only LoF-segments older than a specified threshold. For each gene, we divided all LoF-segments into deciles based on IBD quality score. For instance, segments with IBD quality scores in the tenth decile (which corresponds to the IBD quality score interval [0.47, 1]), strongly suggest the sharing of common ancestors that lived recently and have therefore transmitted extremely recent variation. We then constructed 10 separate LoF-segment burdens, with increasingly more stringent quality score cutoffs (referred as time transformations in our analysis), and performed 10 association tests for each gene, taking the test that resulted in the lowest *p*-value after adjusting significance thresholds by conservatively assuming independence for all tests. Not all genes contained shared LoF-segments for testing, which reduced the total number of tested genes to 14,249. This resulted in a Bonferroni-corrected exome-wide significance threshold of 0.05/(10 × 14,249) for our LoF-segment burden analysis. We note that this approach does not rely on phasing information and that the IBD segment shared with a carrier is equally likely to involve or not the haplotype that harbors the LoF variant. In principle it may be possible to leverage phasing information to increase the accuracy of this approach. Phasing of rare exome sequencing variants is however challenging^80^ and we do not attempt that in this work.

Although gene-based burden tests are meant to implicate specific genes with a known directional effect on the trait, the observed signal may not always be driven by a causal variant, and instead be due to tagging of causal variants in nearby genes. In this case, it is possible that the underlying rare causal variant is tagged by a common variant, which may have been detected in a previous GWAS, leading to synthetic associations – an effect similar to that hypothesized in the context of common variant association^81^. In particular, these common variants may provide better tagging of the underlying true causal variation than our LoF-segment burden score, and would thus remove or significantly reduce the association signal if included as covariates in the test. Based on this principle, for each gene and each trait, we selected up to three genotyped SNPs that were in proximity (±1 Mb from the gene), which were significantly (*p* < 1 × 10^−8^) associated by Loh et al.^82^, and used them as covariates. We observed that this approach often improves the association signal (Supplementary Fig. 20), removing signals that were likely caused by tagging common variants. We refer to analyses that include top associated SNPs as covariates as SNP-adjusted, for either the LoF-segment or WES-LoF burden test; results without the SNP-adjustment are shown in Supplementary Fig. 20 and Supplementary Table 7.

We validated our approaches, both LoF-segment and not SNP-adjusted LoF-segment, which seek to implicitly impute ultra-rare LoF variation between sequenced and non-sequenced individuals, by testing for association between rare variation and seven blood-related phenotypes recently analyzed by Van Hout et al.^49^, and comparing to the results of that same study. However, because summary association statistics for this analysis are not available, we performed our own exome-wide burden testing. Specifically, we used the same testing framework we used in our LoF-segment burden analysis to test for association between phenotypes and burden of LoF variants within a gene in exome-sequenced individuals, adjusting for the same covariates and using the same rank-based inverse normal transformation for the phenotype. We refer to this analysis as WES-LoF burden analysis.

Both LoF-segment burden and WES LoF burden analyses were restricted to unrelated individuals of White British ancestry, as defined by Bycroft et al.^2^, and the LoF-segment burden analysis was further restricted to individuals for which exome sequencing data is not available. This resulted in 303,125 individuals for the two LoF-segment burden tests and 34,422 individuals for the WES LoF. Finally, we note that the UK Biobank has recently released a statement regarding incorrectly mapped variants in the 50k WES Functionally Equivalent (FE) dataset (available online at https://www.ukbiobank.ac.uk/wp-content/uploads/2019/12/Description-of-the-alt-aware-issue-with-UKB-50k-WES-FE-data.pdf.), which we however believe did not introduce any significant biases in our analyses.

Applying our WES-LoF burden analysis, we detected 10 out of 14 exome-wide significant associations also reported by Van Hout et al.^49^. We also detected three additional associations that were not reported by Van Hout et al.^49^: *MAPK8* and *APOC3* with red blood cell distribution width (two-sided *t*-test *p* = 1.33 × 10^−6^ and *p* = 2.13 × 10^−7^ respectively), and *TET2* with eosinophil count (two-sided *t*-test *p* = 1.79 × 10^−8^). Results are summarized in Fig. 5b. These differences are likely ascribed to the slightly different testing strategy we adopted, e.g. the use of a linear model, rather than a linear mixed model, and the exclusion of related samples. Detailed results for these analyses are reported in Supplementary Tables 7 and 8. A QQ-plot verifying the calibration of our test for the SNP-adjusted LoF-segment burden analysis is shown in Supplementary Fig. 26 and, as explained at that point, rare variant stratification is likely to be included in our results^73^ but addressing this issue goes beyond the scope of the current study.

### Reporting summary

Further information on research design is available in the Nature Research Reporting Summary linked to this article.

## Supplementary information

Supplementary Information

Reporting Summary

## Data Availability

The main data items presented in this manuscript (simulation data, UK relatedness matrices, DRC selection annotations, association summary statistics) are available on Zenodo (DOI: 10.5281/zenodo.4012676)^83^. The interactive map with results on population structure is publicly available online at https://ukancestrymap.github.io/. Genomic data sets and annotations analysed in this study include: UK Biobank http://www.ukbiobank.ac.uk/, genetic maps ftp://1000genomes.ebi.ac.uk/vol1/ftp/technical/working/20110106_recombination_hotspots/, gene coordinates http://hgdownload.cse.ucsc.edu/goldenPath/hg19/database/refGene.txt.gz, natural selection annotations ftp://jjwanglab.org/dbPSHP/curation/dbPSHP_20131001.tab, association summary statistics https://data.broadinstitute.org/alkesgroup/UKBB/, allele age estimates http://human.genome.dating, population sizes in UK regions https://www.nomisweb.co.uk/census/2011/qs102ew and https://www.scotlandscensus.gov.uk/ods-web/data-warehouse.html. Data analyses are based on open-source libraries and software programs that are available online: Scipy^79^, Matplotlib^84^, NumPy^85,86^, Pandas^87,88^, Seaborn^89^, BCFtools (http://samtools.github.io/bcftools/), Basemap (https://matplotlib.org/basemap/index.html) and sparseAHC (https://github.com/khabbazian/sparseAHC/).

## Code availability

The FastSMC software is freely available at https://github.com/PalamaraLab/FastSMC.

## References

[1] All of Us Research Program Investigators. The All of Us research program. *N. Engl.**J. Med.* **381**, 668–676 (2019). 10.1056/NEJMsr1809937 PMC8291101 31412182

[2] Bycroft C (2018). The uk biobank resource with deep phenotyping and genomic data. Nature.

[3] Marx V (2015). The DNA of a nation. Nature.

[4] Gaziano JM (2016). Million veteran program: a mega-biobank to study genetic influences on health and disease. J. Clin. Epidemiol..

[5] Kong A (2008). Detection of sharing by descent, long-range phasing and haplotype imputation. Nat. Genet..

[6] Palamara PF, Lencz T, Darvasi A, Pe’er I (2012). Length distributions of identity by descent reveal fine-scale demographic history. Am. J. Hum. Genet..

[7] Palamara PF, Pe’er I (2013). Inference of historical migration rates via haplotype sharing. Bioinformatics.

[8] Ralph P, Coop G (2013). The geography of recent genetic ancestry across europe. PLoS Biol..

[9] Browning SR, Browning BL (2015). Accurate non-parametric estimation of recent effective population size from segments of identity by descent. Am. J. Hum. Genet..

[10] Albrechtsen A, Moltke I, Nielsen R (2010). Natural selection and the distribution of identity-by-descent in the human genome. Genetics.

[11] Gusev A (2011). The architecture of long-range haplotypes shared within and across populations. Mol. Biol. Evol..

[12] Browning SR, Thompson EA (2012). Detecting rare variant associations by identity-by-descent mapping in case-control studies. Genetics.

[13] Gusev A (2011). Dash: a method for identical-by-descent haplotype mapping uncovers association with recent variation. Am. J. Hum. Genet..

[14] Loh P-R, Palamara PF, Price AL (2016). Fast and accurate long-range phasing in a UK Biobank cohort. Nat. Genet..

[15] Loh P-R (2016). Reference-based phasing using the haplotype reference consortium panel. Nat. Genet..

[16] Browning BL, Zhou Y, Browning SR (2018). A one-penny imputed genome from next-generation reference panels. Am. J. Hum. Genet..

[17] Rubinacci, S., Delaneau, O. & Marchini, J. Genotype imputation using the positional burrows wheeler transform. Preprint at https://www.biorxiv.org/content/10.1101/797944v2 (2019). 10.1371/journal.pgen.1009049 PMC7704051 33196638

[18] Marchini J, Howie B (2010). Genotype imputation for genome-wide association studies. Nat. Rev. Genet..

[19] Gusev A (2008). Whole population, genomewide mapping of hidden relatedness. Genome Res..

[20] Browning BL, Browning SR (2013). Improving the accuracy and efficiency of identity-by-descent detection in population data. Genetics.

[21] Naseri, A., Liu, X., Tang, K., Zhang, S. & Zhi, D. Rapid: ultra-fast, powerful, and accurate detection of segments identical by descent (ibd) in biobank-scale cohorts. *Genome Biol.* **20**, 143 (2019). 10.1186/s13059-019-1754-8 PMC6659282 31345249

[22] Wakeley, J. & Wilton, P. R. *Coalescent and Models of Identity By Descent*. Vol. 1, 287–292 (Academic Press, Oxford, 2016).

[23] Palamara, P. F., Terhorst, J., Song, S. & Price, A. L. High-throughput inference of pairwise coalescence times identifies signals of selection and enriched disease heritability. *Nat. Genet.* **50**, 1311–1317 (2018). 10.1038/s41588-018-0177-x PMC6145075 30104759

[24] McVean GA, Cardin NJ (2005). Approximating the coalescent with recombination. Philos. Trans. R. Soc. B Biol. Sci..

[25] Li H, Durbin R (2011). Inference of human population history from individual whole-genome sequences. Nature.

[26] Tataru P, Nirody JA, Song YS (2014). diCal-IBD: demography-aware inference of identity-by-descent tracts in unrelated individuals. Bioinformatics.

[27] Terhorst J, Kamm JA, Song YS (2017). Robust and scalable inference of population history from hundreds of unphased whole genomes. Nat. Genet..

[28] Lawson, D. J., Hellenthal, G., Myers, S. & Falush, D. Inference of population structure using dense haplotype data. *PLoS Genet.* 8, e1002453 (2012). 10.1371/journal.pgen.1002453 PMC3266881 22291602

[29] Leslie S (2015). The fine-scale genetic structure of the british population. Nature.

[30] Haworth S (2019). Apparent latent structure within the uk biobank sample has implications for epidemiological analysis. Nat. Commun..

[31] Edge, M. D. & Coop, G. Attacks on genetic privacy via uploads to genealogical databases. *eLife* **9**, e51810 (2020). 10.7554/eLife.51810 PMC6992384 31908268

[32] Erlich Y, Shor T, Pe’er I, Carmi S (2018). Identity inference of genomic data using long-range familial searches. Science.

[33] Patterson N, Price AL, Reich D (2006). Population structure and eigenanalysis. PLoS Genet..

[34] Yang J (2010). Common snps explain a large proportion of the heritability for human height. Nat. Genet..

[35] Chang CC (2015). Second-generation plink: rising to the challenge of larger and richer datasets. Gigascience.

[36] Jones, B. & Mattingly, D. *An Atlas of Roman Britain: An Atlas of Roman Britain* (Oxbow Books, 1990).

[37] Vandepoele K, Van Roy N, Staes K, Speleman F, van Roy F (2005). A novel gene family NBPF: intricate structure generated by gene duplications during primate evolution. Mol. Biol. Evol..

[38] Barreiro BL, Quintana-Murci L (2009). From evolutionary genetics to human immunology: how selection shapes host defence genes. Nat. Rev. Genet..

[39] Bersaglieri T (2004). Genetic signatures of strong recent positive selection at the lactase gene. Am. J. Hum. Genet..

[40] Fagundes N, M Salzano F, Batzer M, Deininger P, Bonatto S (2005). Worldwide genetic variation at the 3’-utr region of the ldlr gene: possible influence of natural selection. Ann. Hum. Genet..

[41] Stahl PD, Ezekowitz RAB (1998). The mannose receptor is a pattern recognition receptor involved in host defense. Curr. Opin. Immunol..

[42] Buniello, A. et al. The NHGRI-EBI GWAS catalog of published genome-wide association studies, targeted arrays and summary statistics. *Nucleic Acids Res.* **47**, D1005–D1012 (2019). 10.1093/nar/gky1120 PMC6323933 30445434

[43] Hata S (2010). Calpain 8/nCL-2 and Calpain 9/nCL-4 constitute an active protease complex, g-calpain, involved in gastric mucosal defense. PLoS Genet..

[44] Li, Y. et al. Chd1l contributes to cisplatin resistance by upregulating the abcb1-nf-*κ*b axis in human non-small-cell lung cancer. *Cell Death Dis*. **10**, 99 (2019). 10.1038/s41419-019-1371-1 PMC6362241 30718500

[45] Birot A-M (2000). Identification and molecular analysis of banp. Gene.

[46] Raidt, J. et al. Recessive hydin mutations cause primary ciliary dyskinesia without situs abnomalities. *Eur. Respir. J.* **40**, P4808 (2012).

[47] Lines M (2012). Haploinsufficiency of a spliceosomal gtpase encoded by eftud2 causes mandibulofacial dysostosis with microcephaly. Am. J. Hum. Genet..

[48] Palamara P (2015). Leveraging distant relatedness to quantify human mutation and gene-conversion rates. Am. J. Hum. Genet..

[49] Van Hout, C. V., Tachmazidou, I., Backman, J. D. et al. Exome sequencing and characterization of 49,960 individuals in the UK Biobank. *Nature.* **586**, 749–756 (2020). 10.1038/s41586-020-2853-0 PMC7759458 33087929

[50] Consortium GP (2012). An integrated map of genetic variation from 1,092 human genomes. Nature.

[51] Mathieson I, McVean G (2014). Demography and the age of rare variants. PLoS Genet..

[52] Cirulli ET (2020). Genome-wide rare variant analysis for thousands of phenotypes in over 70,000 exomes from two cohorts. Nat. Commun..

[53] Zhao Z (2020). Uk biobank whole-exome sequence binary phenome analysis with robust region-based rare-variant test. Am. J. Hum. Genet..

[54] Kiezun A (2013). Deleterious alleles in the human genome are on average younger than neutral alleles of the same frequency. PLoS Genet.

[55] Albers PK, McVean G (2020). Dating genomic variants and shared ancestry in population-scale sequencing data. PLoS Biol..

[56] McCarthy S (2016). A reference panel of 64,976 haplotypes for genotype imputation. Nat. Genet..

[57] Pritchard JK, Przeworski M (2001). Linkage disequilibrium in humans: models and data. Am. J. Hum. Genet..

[58] Kichaev G (2019). Leveraging polygenic functional enrichment to improve gwas power. Am. J. Hum. Genet..

[59] Astle WJ (2016). The allelic landscape of human blood cell trait variation and links to common complex disease. Cell.

[60] Gieger C (2011). New gene functions in megakaryopoiesis and platelet formation. Nature.

[61] Kanai M (2018). Genetic analysis of quantitative traits in the japanese population links cell types to complex human diseases. Nat. Genet..

[62] Wegmann D (2011). Recombination rates in admixed individuals identified by ancestry-based inference. Nat. Genet..

[63] Hinch AG (2011). The landscape of recombination in african americans. Nat. Genet..

[64] Tian X, Browning BL, Browning SR (2019). Estimating the genome-wide mutation rate with three-way identity by descent. Am. J. Hum. Genet..

[65] Gusev A (2012). Low-pass genome-wide sequencing and variant inference using identity-by-descent in an isolated human population. Genetics.

[66] Shemirani, R. et al. Rapid detection of identity-by-descent tracts for mega-scale datasets. Preprint at https://www.biorxiv.org/content/10.1101/749507v1 (2019). 10.1038/s41467-021-22910-w PMC8192555 34112768

[67] Zhou, Y., Browning, S. R. & Browning, B. L. A fast and simple method for detecting identity by descent segments in large-scale data. *Am. J. Hum. Genet.* **106**, 426–437 (2020). 10.1016/j.ajhg.2020.02.010 PMC7118582 32169169

[68] Durbin R (2014). Efficient haplotype matching and storage using the positional burrows–wheeler transform (pbwt). Bioinformatics.

[69] Minichiello MJ, Durbin R (2006). Mapping trait loci by use of inferred ancestral recombination graphs. Am. J. Hum. Genet..

[70] Rasmussen MD, Hubisz MJ, Gronau I, Siepel A (2014). Genome-wide inference of ancestral recombination graphs. PLoS Genet..

[71] Kelleher J (2019). Inferring whole-genome histories in large population datasets. Nat. Genet..

[72] Speidel L, Forest M, Shi S, Myers SR (2019). A method for genome-wide genealogy estimation for thousands of samples. Nat. Genet..

[73] Mathieson I, McVean G (2012). Differential confounding of rare and common variants in spatially structured populations. Nat. Genet..

[74] Hobolth A, Christensen OF, Mailund T, Schierup MH (2007). Genomic relationships and speciation times of human, chimpanzee, and gorilla inferred from a coalescent hidden markov model. PLoS Genet..

[75] Simonsen K, Churchill G (1997). A markov chain model of coalescence with recombination. Theor. Popul. Biol..

[76] Hobolth A, Jensen JL (2014). Markovian approximation to the finite loci coalescent with recombination along multiple sequences. Theor. Popul. Biol..

[77] Palamara PF (2016). ARGON: fast, whole-genome simulation of the discrete time wright-fisher process. Bioinformatics.

[78] Manichaikul A (2010). Robust relationship inference in genome-wide association studies. Bioinformatics.

[79] Virtanen P (2020). SciPy 1.0: fundamental algorithms for scientific computing in python. Nat. Methods.

[80] Choi Y, Chan AP, Kirkness E, Telenti A, Schork NJ (2018). Comparison of phasing strategies for whole human genomes. PLoS Genet..

[81] Dickson SP, Wang K, Krantz I, Hakonarson H, Goldstein DB (2010). Rare variants create synthetic genome-wide associations. PLoS Biol..

[82] Loh P-R, Kichaev G, Gazal S, Schoech AP, Price AL (2018). Mixed-model association for biobank-scale datasets. Nat. Genet..

[83] Nait Saada, J. et al. Identity-by-descent detection across 487,409 British samples reveals fine scale population structure and ultra-rare variant associations: data related to publication (2020). 10.1038/s41467-020-19588-x PMC7704644 33257650

[84] Hunter JD (2007). Matplotlib: a 2d graphics environment. Comput. Sci. Eng..

[85] Oliphant, T. E. *A guide to NumPy*, Vol. 1 (Trelgol Publishing USA, 2006).

[86] Van Der Walt S, Colbert SC, Varoquaux G (2011). The numpy array: a structure for efficient numerical computation. Comput. Sci. Eng..

[87] The pandas development team. pandas-dev/pandas: Pandas (2020).

[88] Wes, M. Data Structures for Statistical Computing in Python. In *Proceedings of the 9th Python in Science Conference*, 56–61 (2010).

[89] Waskom, M. et al. mwaskom/seaborn: v0.8.1 (September, 2017).

