# Supplementary material for: Identity-by-descent detection across 487,409 British samples reveals fine scale population structure and ultra-rare variant associations
Source: Nat Commun. 2020 Nov 30;11:6130. doi: 10.1038/s41467-020-19588-x (PMC7704644; doi:10.1038/s41467-020-19588-x)
Supplement: Supplementary file 1 — Supplementary Information [file 41467_2020_19588_MOESM1_ESM.pdf]

Supplementary Information

Identity-by-descent detection across 487,409 British samples reveals fine scale population structure and ultra-rare variant associations

Nait Saada et al.

Supplementary Figures

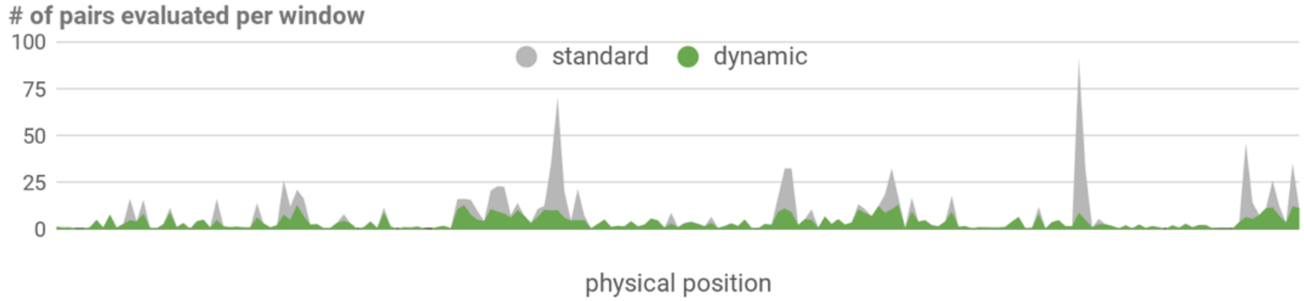

Supplementary Fig. 1. GERMLINE and GERMLINE2 comparison. Search complexity for standard vs dynamic hashing. Number of pairs of individuals (in millions) with identical haplotypes in each genome window using GERMLINE standard hash (gray) and the proposed GERMLINE2 dynamic hash (green). Analysis of $16,000$ random UK Biobank samples from chromosome 22. Multiple regions of the genome exhibit sharing between more than 10% of all pairs, requiring extensive follow-up analysis in standard mode.

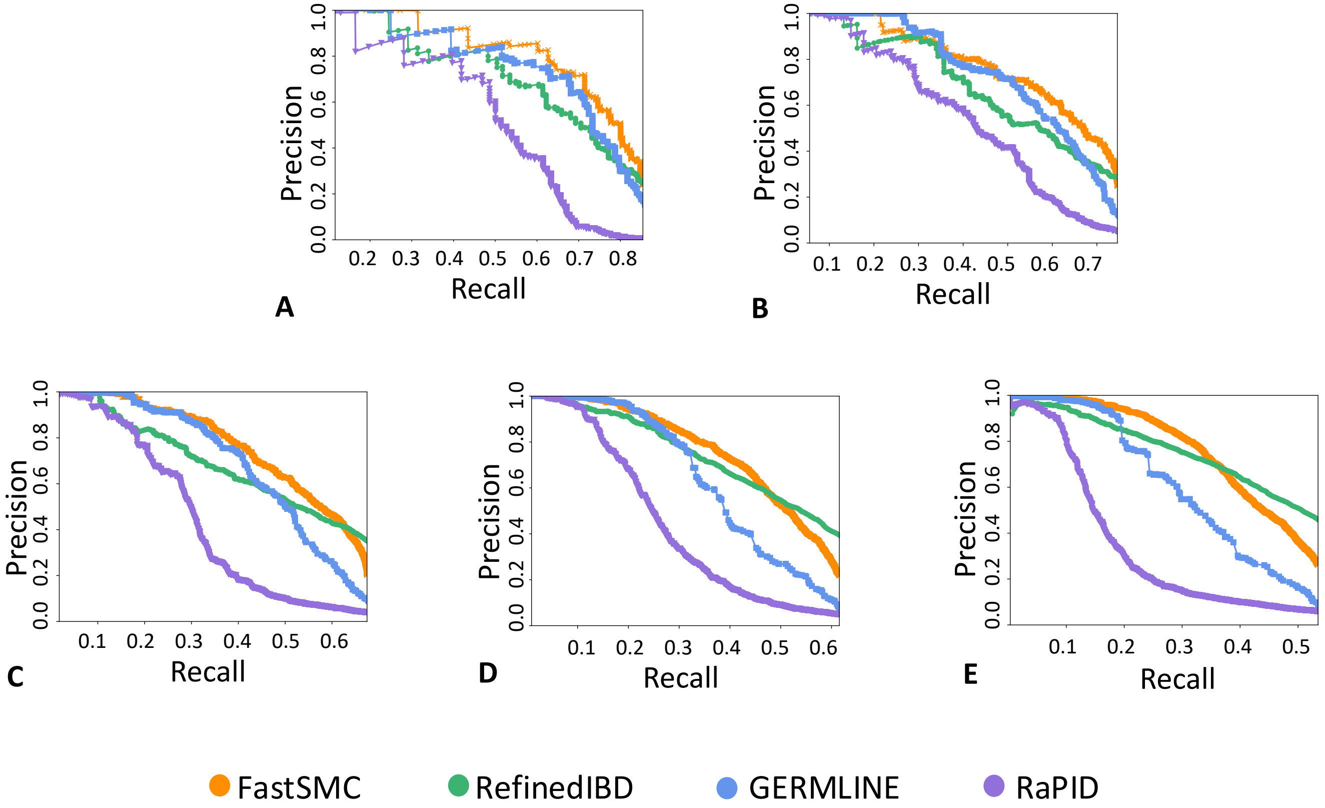

Supplementary Fig. 2. Accuracy evaluation for IBD detection at different time scales. Precision-recall curves within the common recall range (i.e where all methods are able to provide predictions) in the past 25 (A), 50 (B), 100 (C), 150 (D) and 200 (E) generations, randomly sampled from 10 realistic simulated datasets. Each dataset consists of a 30Mb chromosome under European demographic history model, recombination rates from a human chromosome 2 and SNP ascertainment matching UKBB allele frequencies. Precision refers to the fraction of true IBD segments among the retrieved segments while recall measures the proportion of actual IBD segments that are correctly identified as such. For each method (FastSMC, RefinedIBD [1], GERMLINE [2] and RaPID [3]), optimal parameters from the grid search were used.

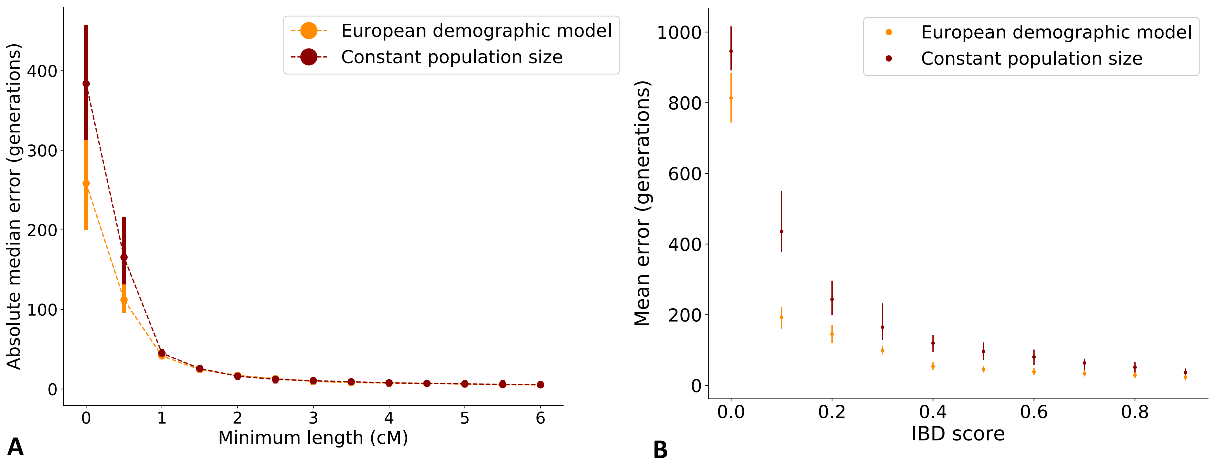

Supplementary Fig. 3. Demographic model misspecification. Effects of demographic model misspecification in age estimate of IBD segments. We simulated 10 batches of 300 haploid samples from the first 30Mb of a human chromosome 2 and a constant population size of $10,000$ diploid individuals, and 10 realistic batches according to the setup described in Methods. We ran FastSMC on both datasets, assuming a European demographic model, with a time threshold of 50 generations and a minimum length of 0.001 cM. We report (A) the absolute median error between the true age and the MAP age estimate while varying the minimum length of the IBD segments, and (B) the mean error between the true age and the MAP estimate while varying the minimum IBD score. Vertical lines represent the mean values +/- SEM over all 10 batches in both (A) and (B). Misspecifying the demographic model results in biased age estimates for very short ($<$ 1cM) or intermediate IBD score segments.

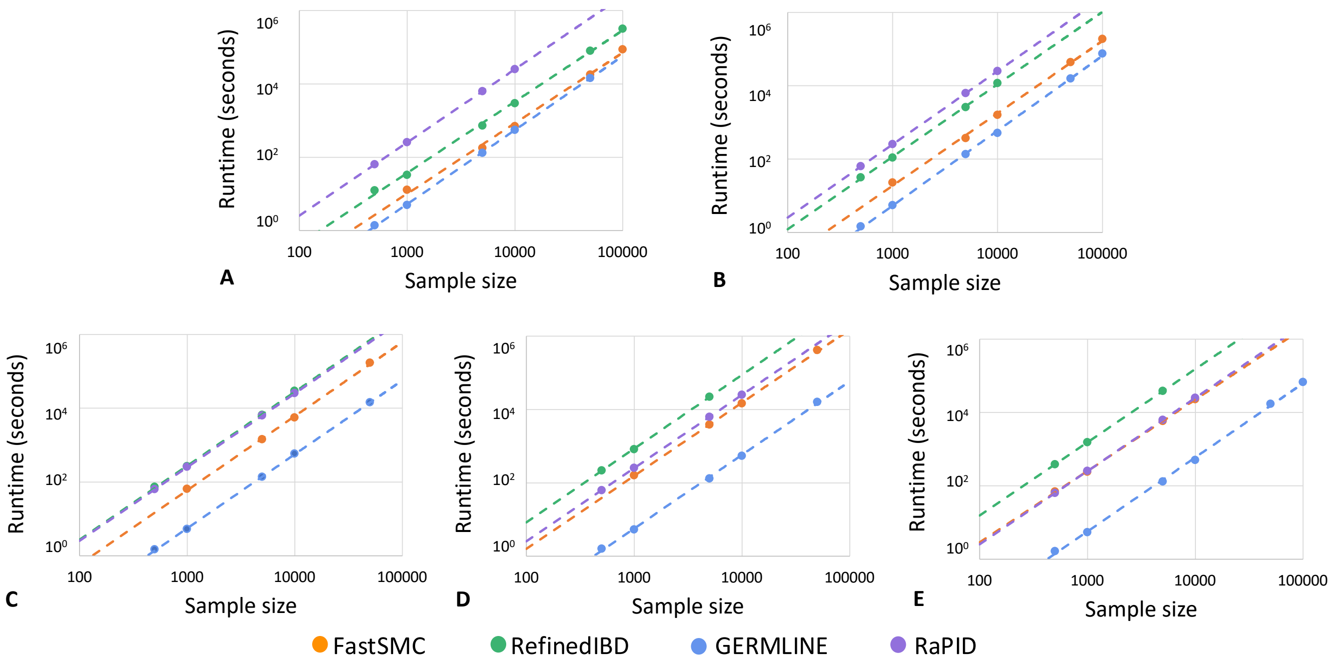

Supplementary Fig. 4. Running time evaluation for IBD detection at different time scales. Running time (CPU seconds) using chromosome 20 of the UKBB across 7,913 SNPs, for IBD segments detection within the past 25 (A), 50 (B), 100 (C), 150 (D) and 200 (E) generations. The complete cohort of 487,409 samples from the UKBB was randomly downsampled into batches. Only one thread was used for each method (FastSMC, RefinedIBD, GERMLINE and RaPID) and parameters from grid search were used. Trend lines in logarithmic scale reflect differences in the quadratic components of each algorithm.

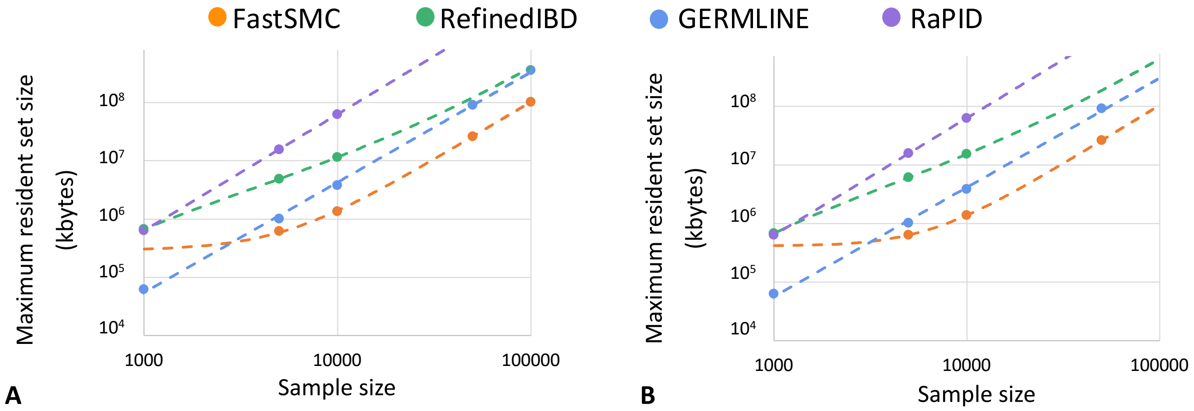

Supplementary Fig. 5. Memory usage for IBD detection at different time scales. Memory usage in kilobytes of FastSMC, RefinedIBD, GERMLINE and RaPID for IBD detection within the past 50 (A) and 100 (B) generations, with optimal parameter values from the grid search across 7,913 SNPs (chromosome 20). The complete cohort of 487,409 samples from the UKBB was randomly downsampled into batches.

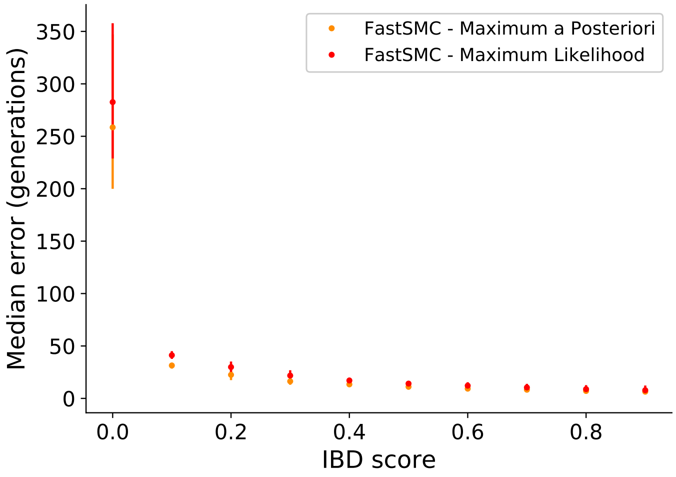

Supplementary Fig. 6. MAP and MLE age estimates comparison. Absolute median error in age estimation in FastSMC for both the MAP and the MLE estimates, at different IBD score thresholds. Data are presented as mean values with 95% confidence intervals over 10 different simulations (each of them consisting of a 30Mb chromosome under European demographic history model, recombination rates from a human chromosome 2 and SNP ascertainment matching UKBB allele frequencies).

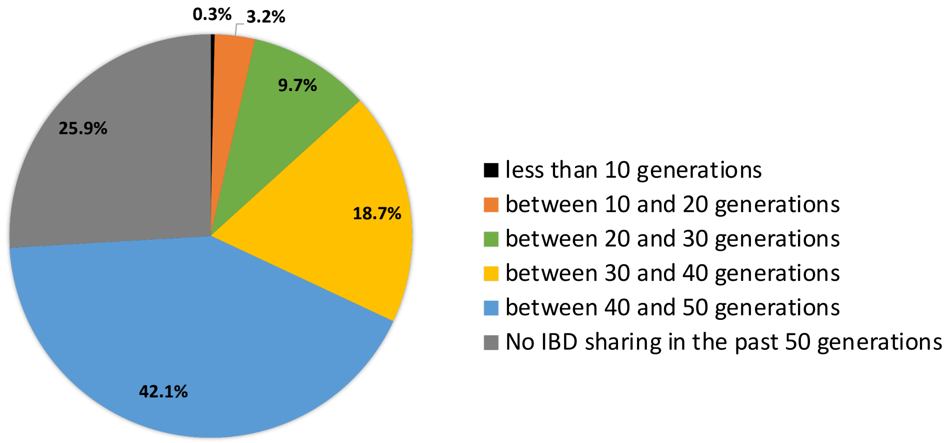

Supplementary Fig. 7. IBD sharing among pairs of samples in the UK Biobank dataset. For each pair of individuals (out of 118, 783, 522, 936 total pairs) in the UK Biobank cohort (487, 409 diploid samples) we determined the most recent estimated shared segment, which provides an upper bound for the pair's genealogical relationship. 74% of all pairs of British individuals were estimated to share common ancestry at some point within the past 50 generations.

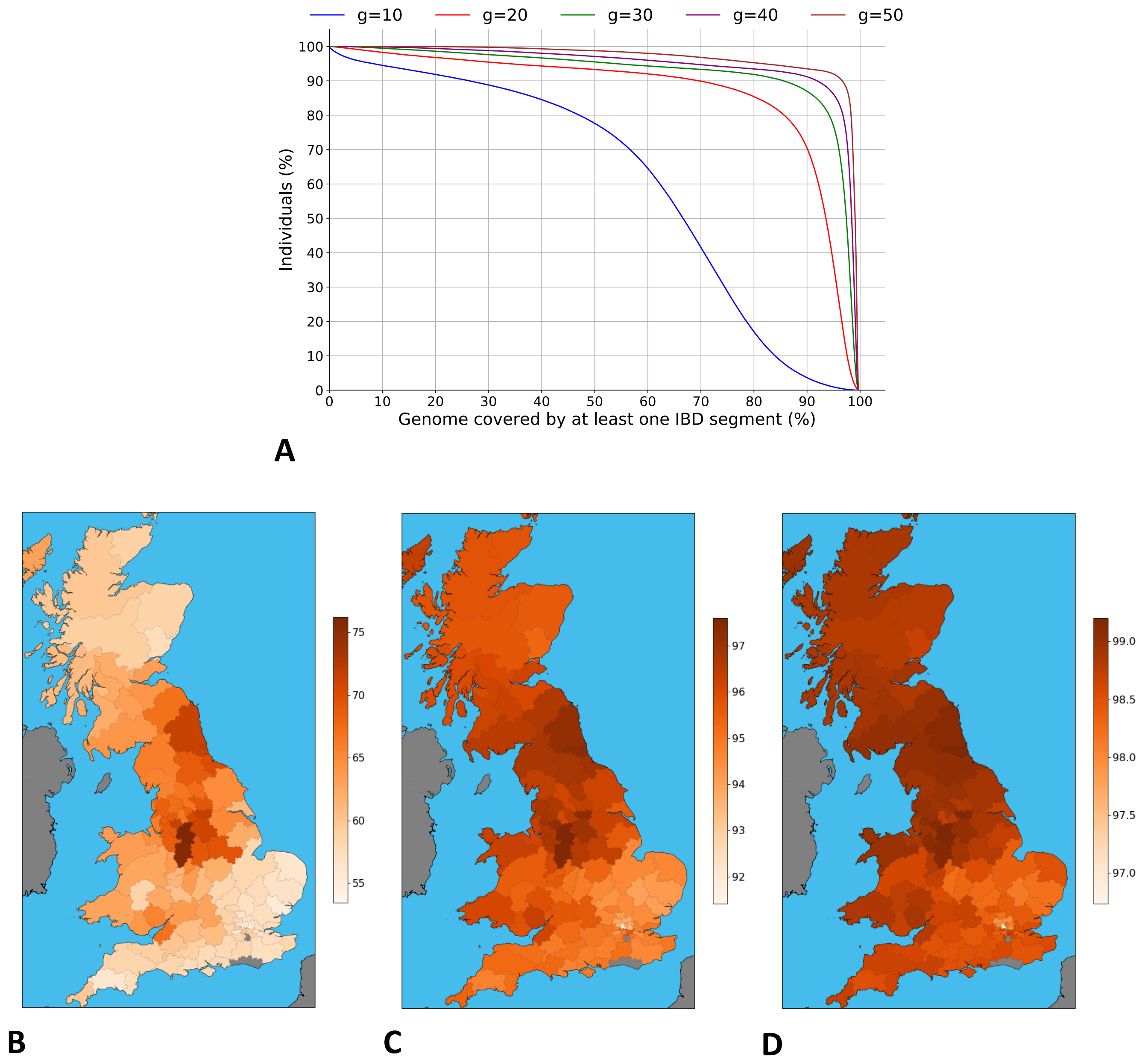

Supplementary Fig. 8. Fraction of genome covered by IBD segments in the UK Biobank dataset. A. Fraction of genome covered by at least one IBD segment (%) for 487, 409 samples from the UK Biobank cohort within the past 10, 20, 30, 40 and 50 generations. B, C, D. Average fraction of genome covered by at least one IBD segment (%) within UK postcodes for 432, 968 samples with known geographic coordinats from the UK Biobank cohort in the past 10 (B), 30 (C) and 50 (D) generations. Regions with no data available are coloured in gray.

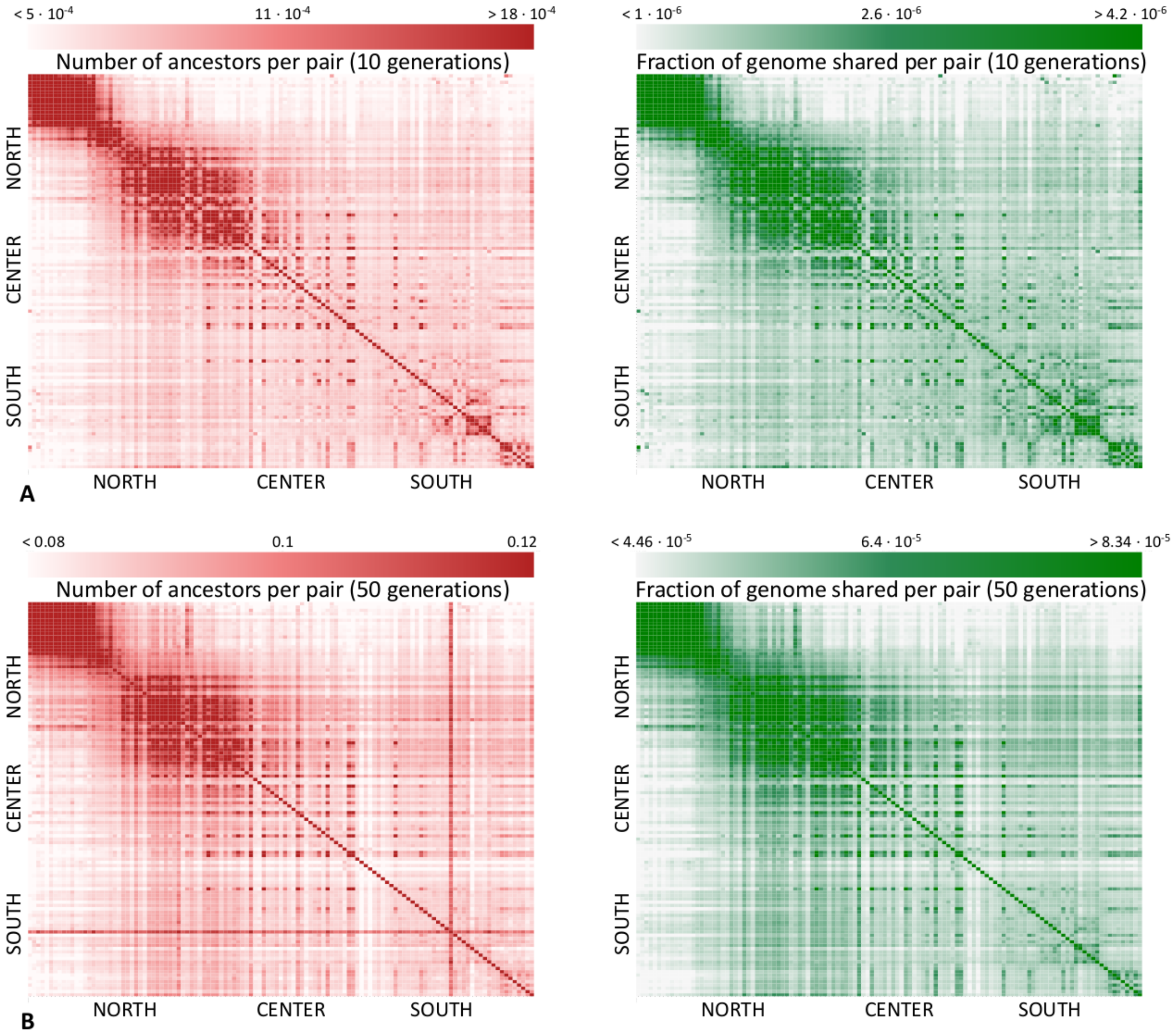

Supplementary Fig. 9. IBD sharing in the past 10 and 50 generations across UK postcodes in the UK Biobank dataset. Average number of IBD segments (left) and fraction of genome (right) shared per pair of individuals across all pair of postcodes in the UK (excluding Northern Ireland) in the past 10 (A) and 50 (B) generations. Segments with IBD score smaller than 0.4 were excluded, resulting approximately in recall of 0.6 and precision of 0.8 based on simulations. The red cross appearing in the South of the UK in B corresponds to Sutton postcode area (covering south-west London), a cosmopolitan region where samples share many ancestors but small fraction of genome with the rest of the country, revealing deep ancestral ties (i.e short IBD segments) throughout the UK.

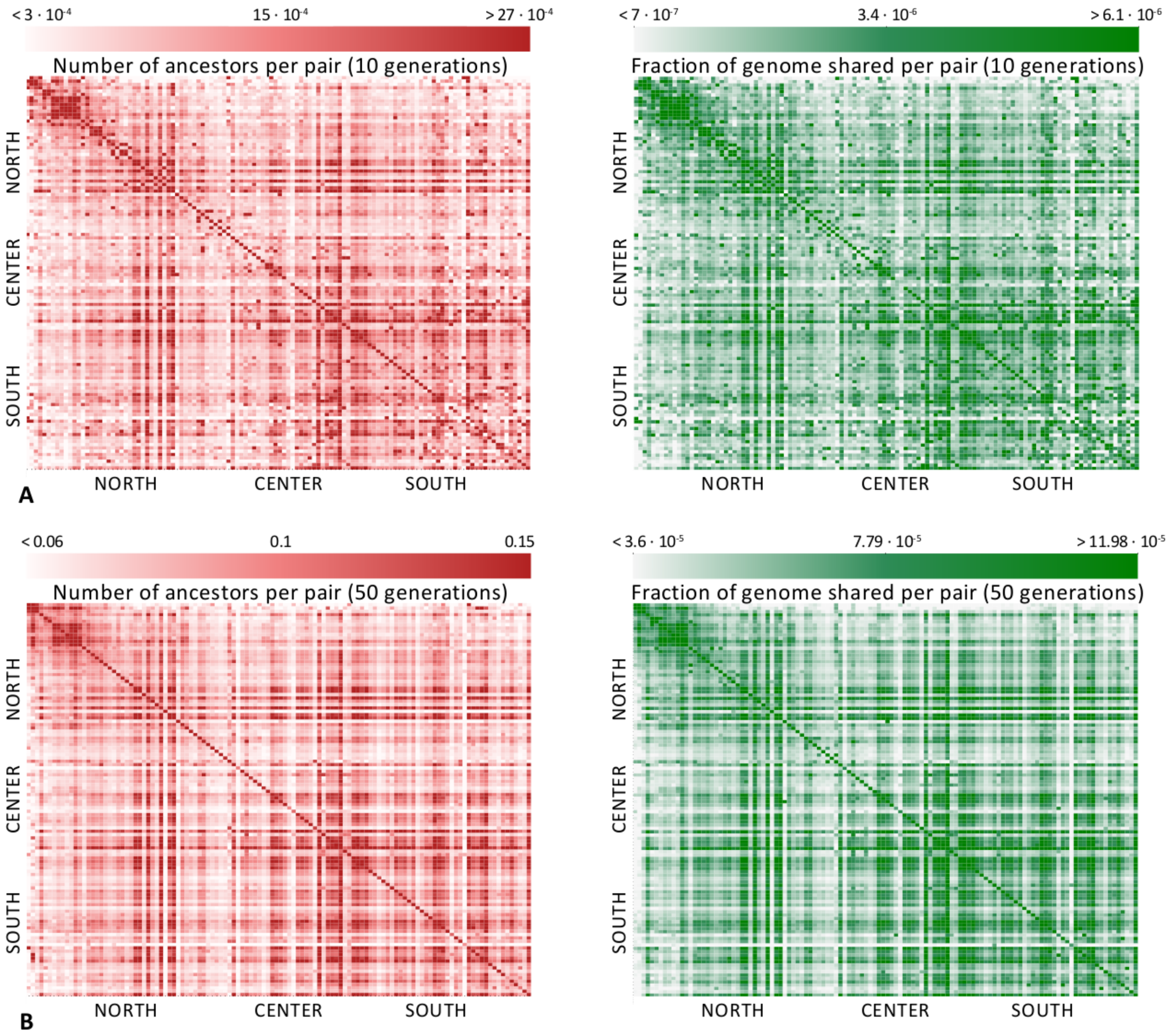

Supplementary Fig. 10. IBD sharing in the past 10 and 50 generations across UK postcodes for non-White British samples in the UK Biobank dataset. Average number of IBD segments (left) and fraction of genome (right) shared per pair of individuals across all pair of postcodes in the UK (excluding Northern Ireland) in the past 10 (A) and 50 (B) generations. Segments with IBD score smaller than 0.4 were excluded, resulting approximately in recall of 0.6 and precision of 0.8 based on simulations. White British samples as defined by Bycroft et al. [4] were excluded, resulting in 38, 801 individuals with available birth location. Results reveal similar patterns with the ones observed for the whole UK Biobank cohort.

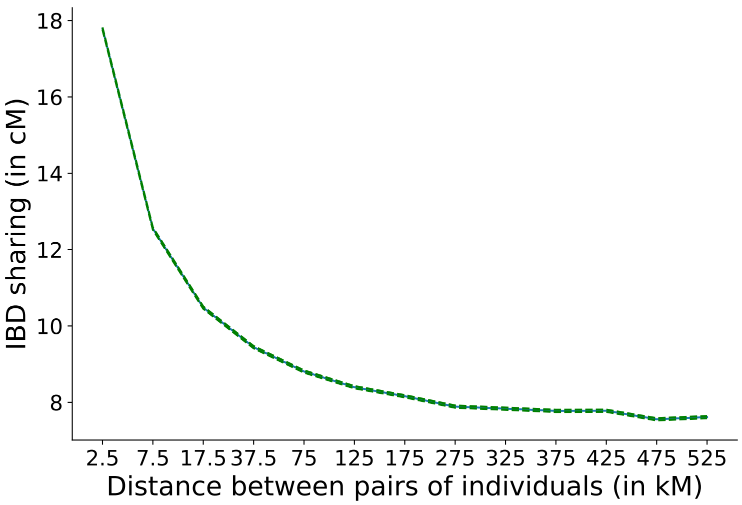

Supplementary Fig. 11. IBD sharing and geographic distance. We randomly sampled 5,000,000 pairs from the UKBB cohort and computed the IBD sharing in cM (i.e sum of IBD segments lengths along the genome within the past 10 generations) for each of them. We partitioned these pairs depending on the distance in kilometers (kM) in the birth locations of the two individuals (less than 5kM, between 5 and 10kM, between 10 and 25kM, between 25 and 50kM, and then every 50kM up to 500kM). The blue line represents the average IBD sharing across all pairs of random samples, and the green trend lines correspond to the standard error of the mean across all pairs (assuming pairs of individuals are independent, which is approximately true when looking at sharing of very recent segments). We observe strong correlation between genetic and geographic distance, demonstrating that close relatives tend to be geographically clustered.

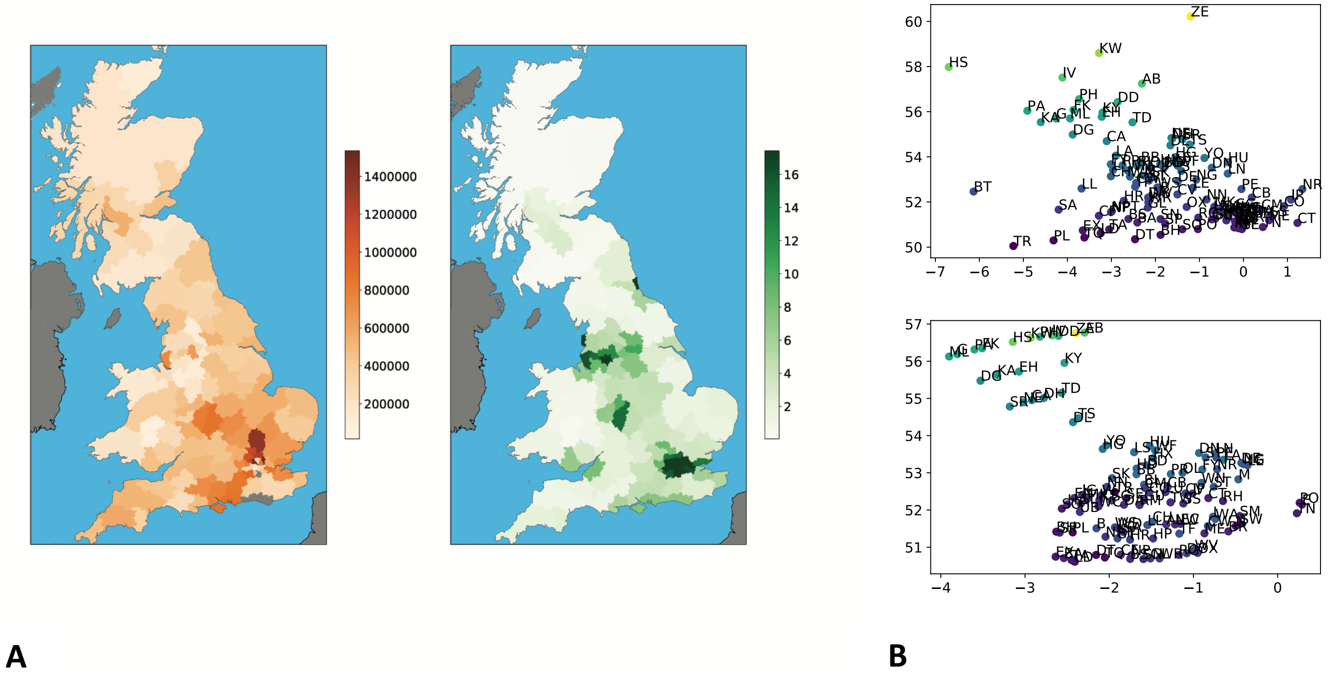

Supplementary Fig. 12. Estimation of effective size and reconstruction of physical distances with IBD sharing. A. Recent effective population size estimation based on IBD sharing in the past 10 generations (left) and 2011 census population size (number of persons per hectare) within postcodes (right). Census population data comes from Nomis web-based dataset for England and Wales, and from the Data Warehouse for Scotland (data only available on the area level, we computed estimates for postcodes). Regions with no data available are coloured in gray. B. Real physical distances between postcodes (top) and isomap projections using IBD sharing within the past 600 years (bottom), x-axis is longitude and y-axis is latitude. The isomap projection was obtained on the IBD sharing postcode dissimilarity matrix and by considering 8 nearest neighbours. We then applied affine transformations (rotation, translation and scaling) to minimize the root mean squared of the reconstruction error (RMSE) [5]. The RMSE obtained is 179 km, 95% CI=[163,196].

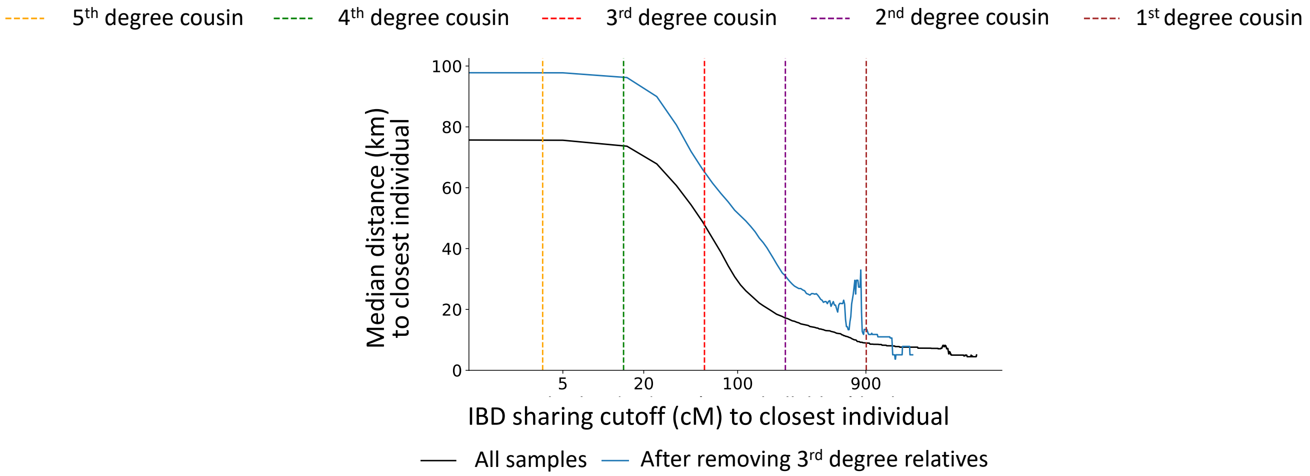

Supplementary Fig. 13. Genetic relatedness and geographic distances (home address) in the UK Biobank dataset, before and after removing related individuals. For each UK Biobank sample with available home address, we detected the individual sharing the largest total amount (in cM) of genome IBD within the past 10 generations (referred to as closest individual). For each value x of total shared genome (in cM) on the X-axis, we report the median distance (km, computed every 10 cM) for all pairs of (sample, closest individual) who shared at least x. Vertical dashed lines indicate the expected value of the total IBD sharing for k-th degree cousins, computed using $2G(1/2)^{2(k+1)}$, where $G = 7247.14$ is the total diploid genome size (in cM) and k represents the degree of cousin relationship (e.g. $k = 2$ for second degree cousins, separated by $2(k + 1)$ generations) [6]. We show results obtained using either all individuals with available home address data ($N = 482,832$; black line), or all individuals with available home address data after removing ≤3rd degree relatives (e.g. first degree cousins), detected by Bycroft et al. [4] using the KING software [7] ($N = 403,356$; blue line). After excluding ≤3rd degree relatives, the sample size for high IBD sharing (≥700 cM) becomes small (≤30, compared to ≤30,000 without excluding any sample), making the estimate noisy. The peak observed for large values of IBD sharing is thus likely due to a small number of related groups.

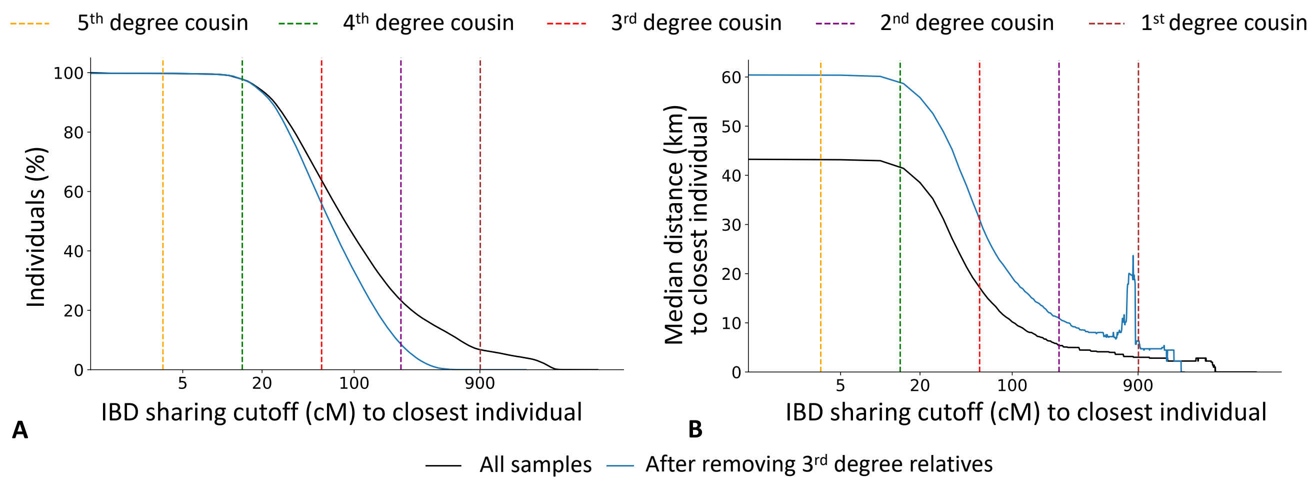

Supplementary Fig. 14. Genetic relatedness and geographic distances (birth locations) in the UK Biobank dataset, before and after removing related individuals. For each UK Biobank sample with available geographic data, we detected the individual sharing the largest total amount (in cM) of genome IBD within the past 10 generations (referred to as closest individual). A. For each value x of total shared genome (in cM) on the X-axis, we report the percentage of UK Biobank samples (Y-axis) that share x or more with their closest individual. B. For each value x of total shared genome (in cM) on the X-axis, we report the median distance (km, computed every 10 cM) for all pairs of (sample, closest individual) who shared at least x. Vertical dashed lines indicate the expected value of the total IBD sharing for k-th degree cousins, computed using $2G(1/2)^{2(k+1)}$, where $G = 7247.14$ is the total diploid genome size (in cM) and k represents the degree of cousin relationship (e.g. $k = 2$ for second degree cousins, separated by $2(k+1)$ generations) [6]. We show results obtained using either all White British (A) and non-White British (B) individuals with available birth location data ($N = 432,968$; black lines), or all White British (A) and non-White British (B) individuals with available geographic data after removing ≤3rd degree relatives (e.g. first degree cousins), detected by Bycroft et al. [4] using the KING software [7] ($N = 357,588$; blue lines). After excluding ≤3rd degree relatives, the sample size for high IBD sharing (≥700 cM) becomes small (≤30, compared to ≤30,000 without excluding any sample), making the estimate noisy. The peak observed for large values of IBD sharing is thus likely due to a small number of related groups.

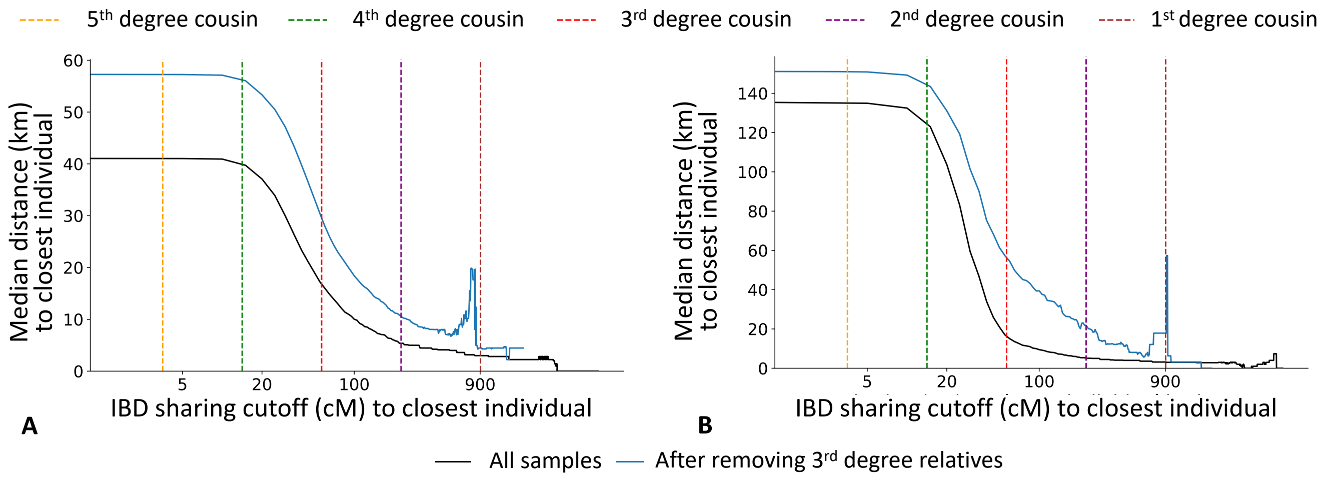

Supplementary Fig. 15. Genetic relatedness and geographic distances (birth location) in the UK Biobank dataset, before and after removing related individuals, for White British and non-White British samples. For each UK Biobank sample with available birth location among White British individuals (A) or non-White British individuals (B), we detected the individual sharing the largest total amount (in cM) of genome IBD within the past 10 generations (referred to as closest individual). For each value x of total shared genome (in cM) on the X-axis, we report the median distance (km, computed every 10 cM) for all pairs of (sample, closest individual) who shared at least x. Vertical dashed lines indicate the expected value of the total IBD sharing for k-th degree cousins, computed using $2G(1/2)^{2(k+1)}$, where $G = 7247.14$ is the total diploid genome size (in cM) and k represents the degree of cousin relationship (e.g. $k = 2$ for second degree cousins, separated by $2(k+1)$ generations) [6]. We show results obtained using either all White British (A) and non-White British (B) individuals with available birth location data ($N = 394,167$ in A, $N = 38,801$ in B; black lines), or all White British (A) and non-White British (B) individuals with available geographic data after removing ≤3rd degree relatives (e.g. first degree cousins), detected by Bycroft et al. [4] using the KING software [7] ($N = 324,767$ in A, $N = 32,821$ in B; blue lines). A. Only considering White British samples results in a median error of 42km for any random White British sample (compared to 45km when considering all samples) while the median error when taking random pairs of White British samples (irrespective of IBD sharing) is 199.2km (95% CI=[198.9,199.4]). B. Only considering non-White British samples results in a median error for any random non-White British sample of 135km, while the median error when taking random pairs of non-White British samples (irrespective of IBD sharing) is 211.9km (95% CI=[211.2, 212.2]). After excluding ≤3rd degree relatives, the sample size for high IBD sharing (≥700 cM) becomes small, making the estimate noisy. The peak observed for large values of IBD sharing is thus likely due to a small number of related groups.

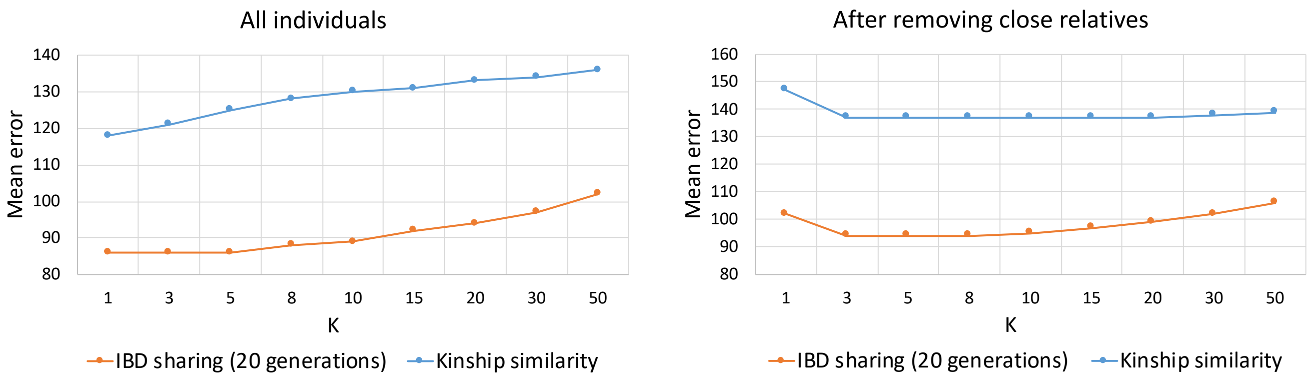

Supplementary Fig. 16. Fine-tuning of the K-nearest neighbours algorithm. (left) Average error when predicting birth location of 10,000 random samples from the UKBB, applying the K-nearest-neighbours algorithm while varying the value of the parameter K, using both IBD sharing among individuals within the past 20 generations and kinship similarity. (right) Average error of 8,226 random samples from the UKBB, after excluding close relatives ($\leq$3rd degree relatives), using the same procedure.

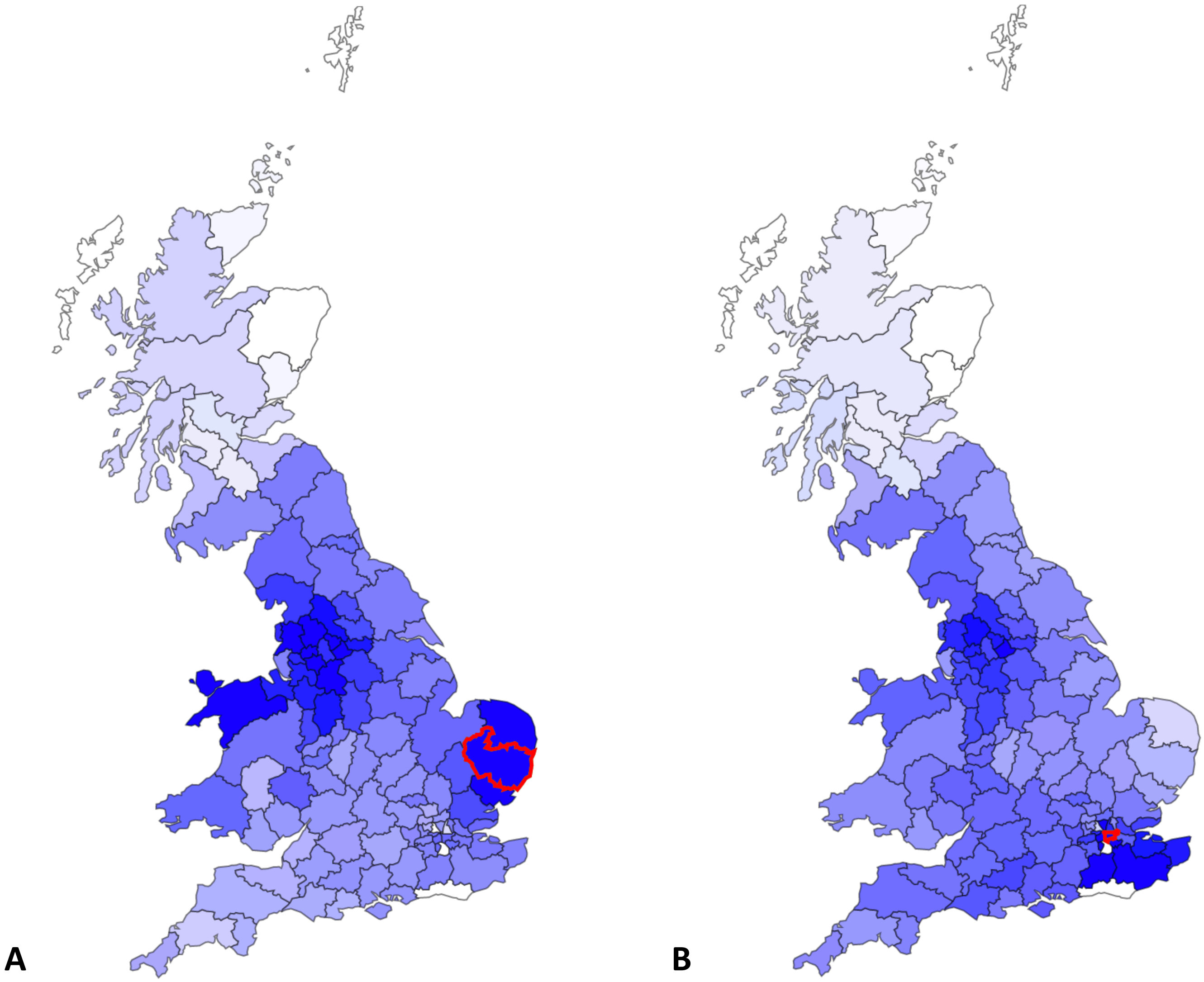

Supplementary Fig. 17. Pervasive IBD sharing with North-West England. Individuals throughout the UK, including individuals from cosmopolitan regions such as London, have deep genetic relationship with modern-day individuals from North-West England, in addition to nearby regions. This figure displays the average fraction of genome shared through IBD segments in the past 1,500 years per pair of individuals between the IP postcode (corresponding to Ipswich, in red) and other UK regions (A), and between the SE postcode (corresponding to South East London, in red) and other UK regions (B). The darker the color is, the higher the average fraction of genome shared is. More details and an interactive map can be found at https://ukancestrymap.github.io/

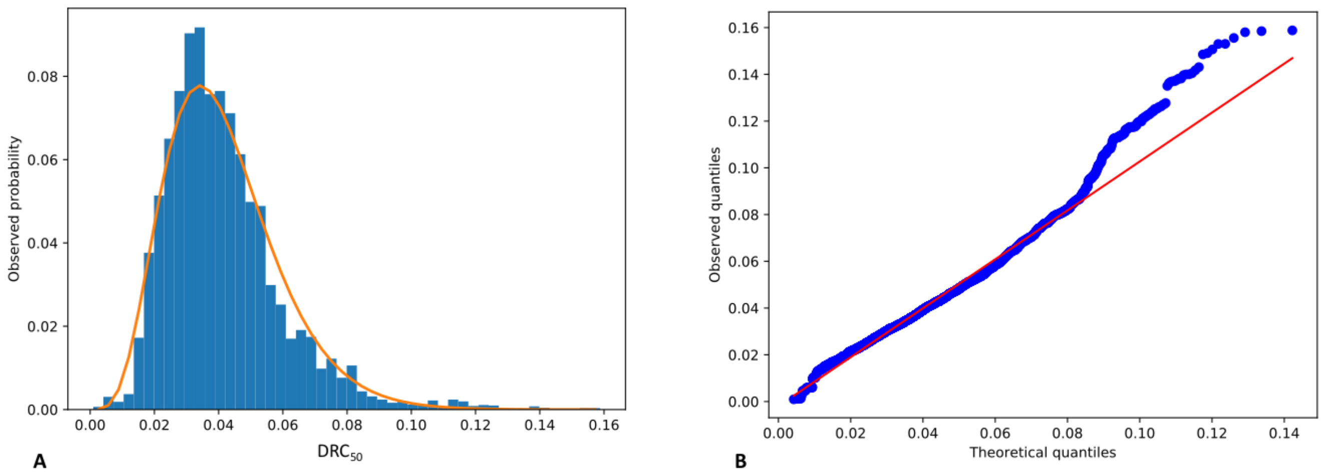

Supplementary Fig. 18. Empirical null model for the DRC_{50} statistic. Empirical null model for detection of recent positive selection, fitted using a Gamma distribution with shape, location and scale parameters. A. Empirical distribution (in blue) and Gamma-fit (orange curve) for the DRC_{50} statistic in the putative neutral regions of the genome in the UKBB, after excluding significant loci falling within these putative neutral regions (9, 165 observations from 0.05 cM windows). B. Quantile-quantile plot for the DRC_{50} statistic in the putative neutral regions of the genome in the UKBB, after excluding significant loci falling within these putative neutral regions (9, 165 observations from 0.05 cM windows).

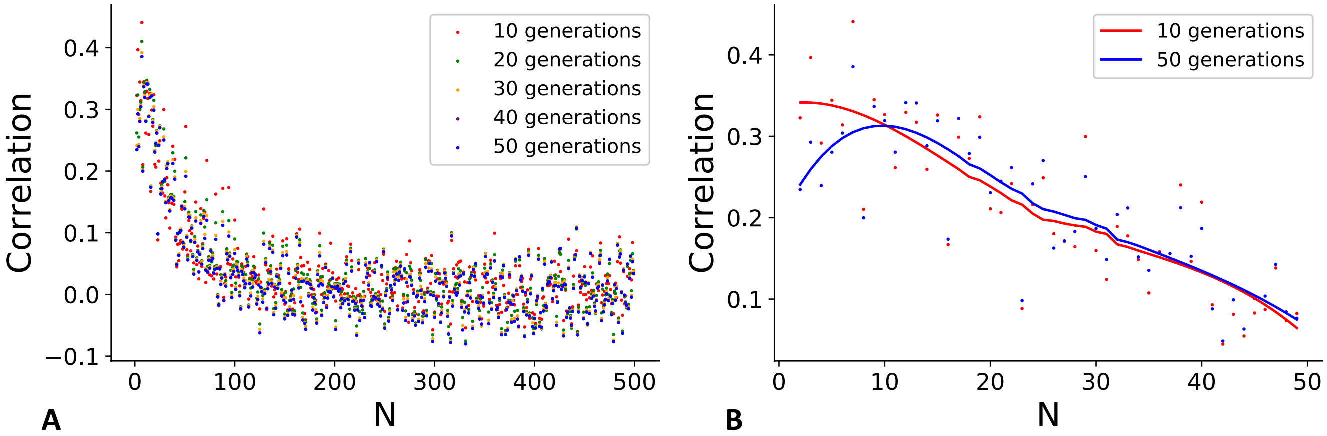

Supplementary Fig. 19. Correlation between IBD sharing and sharing of rare variants. A. Correlation between IBD sharing (average number of IBD segments per pair within UK regions in the past 10, 20, 30, 40 and 50 generations in the UK Biobank cohort) and ultra-rare variants sharing (average number of F_N mutations per pair within UK regions in the UK Biobank 50k Exome Sequencing Data Release, for any N between 2 and 499). B. Non-parametric regression of the correlation between IBD sharing (average number of IBD segments per pair within UK regions in the past 10 and 50 generations in 487,409 samples in the UK Biobank dataset) and ultra-rare variants sharing (average number of F_N mutations per pair within UK regions in the UK Biobank 50k Exome Sequencing Data Release, for values of N smaller than 50).

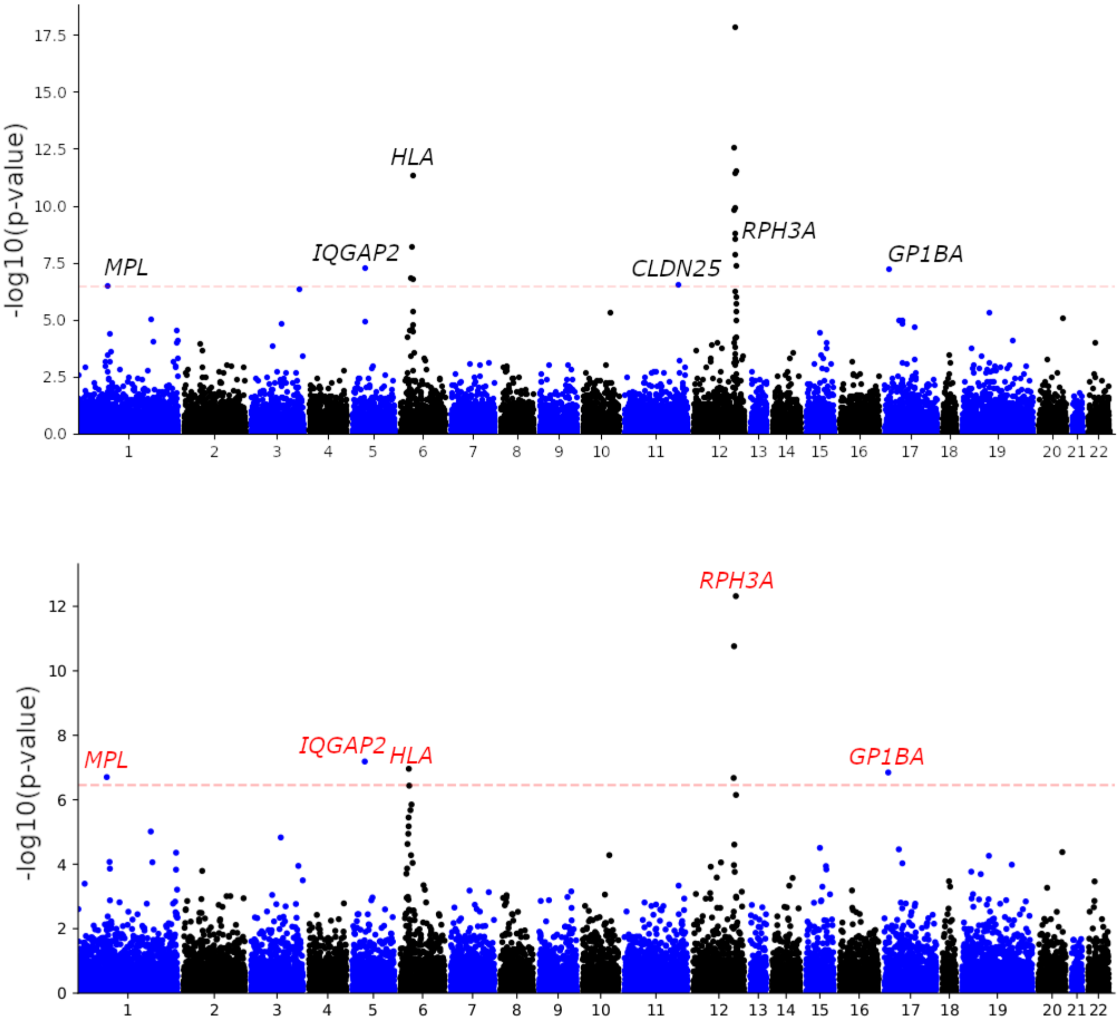

Supplementary Fig. 20. LoF-segment burden exome-wide Manhattan plots for platelet count with and without SNP-adjustment. Labelled genes are exome-wide significant after adjusting for multiple testing ($p < 0.05/(14,249 \times 10) = 3.51 \times 10^{-7}$; dashed red line). We compare results before (top) and after (bottom) adjusting for common SNP associations. Both LoF-segment burden analyses used 303,125 UK Biobank samples not included in the exome sequencing cohort. The cluster of genes in chromosome 12 labeled as *RPH3A* (the top association) contains *KCTD10, TCHP* and *RPH3A* and the signal with *CLDN25* was cleared after SNP-adjustment. Red labels in the lower plot indicate associations that were not detected in our WES-based LoF burden analysis or reported by Van Hout et al. [8]. Exact p-values are reported in Supplementary Table 7.

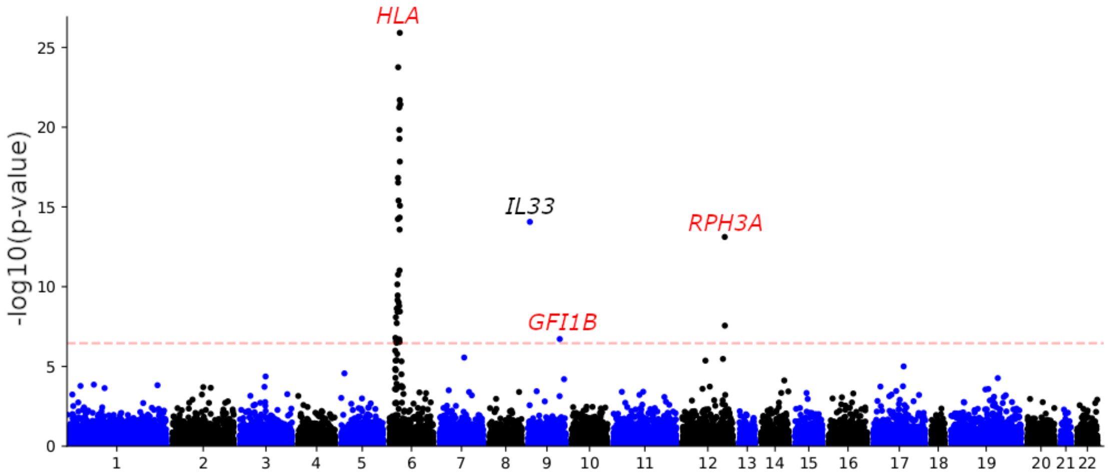

Supplementary Fig. 21. LoF-segment burden exome-wide Manhattan plot for eosinophill count. Labelled genes are exome-wide significant (after adjusting for multiple testing, $p < 0.05/(14,249 \times 10) = 3.51 \times 10^{-7}$; dashed red line). The LoF-segment burden analysis (with SNP adjustment) used 303,125 UK Biobank samples not included in the exome sequencing cohort. We identified one locus previously reported by Van Hout et al. [8] (black label), and additional loci on chromosomes 6,9 and 12 (labels in red). Table 2 reports the list of genes within the gene cluster labeled as HLA. Exact p-values are reported in Supplementary Table 7.

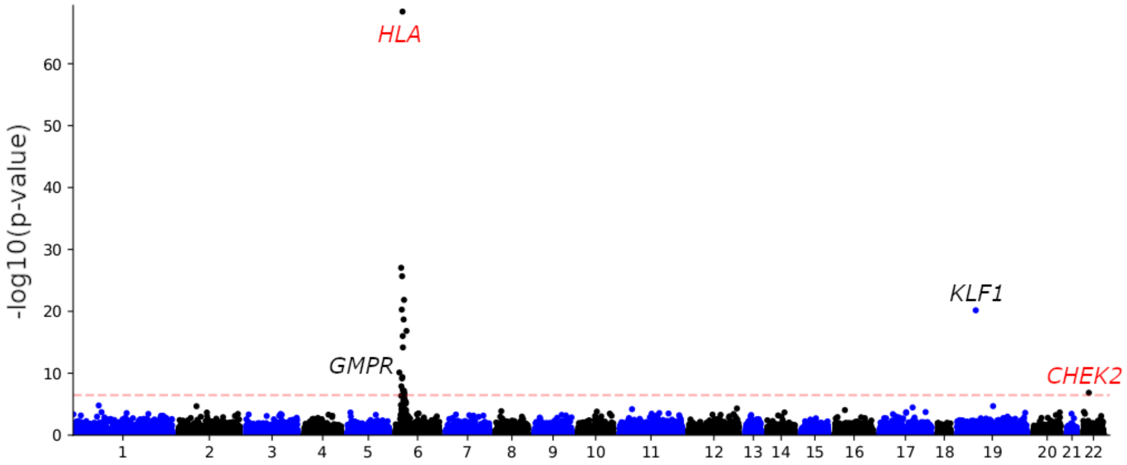

Supplementary Fig. 22. LoF-segment burden exome-wide Manhattan plot for mean corpuscular haemoglobin. Labelled genes are exome-wide significant (after adjusting for multiple testing, $p < 0.05/(14,249 \times 10) = 3.51 \times 10^{-7}$; dashed red line). The LoF-segment burden analysis (with SNP adjustment) used 303,125 UK Biobank samples not included in the exome sequencing cohort. We identified two loci previously reported by Van Hout et al. [8], *KLF1* and *GMPR* (gene labels in black), and two novel associations at HLA and *CHEK2* (labels in red). Exact p-values are reported in Supplementary Table 7.

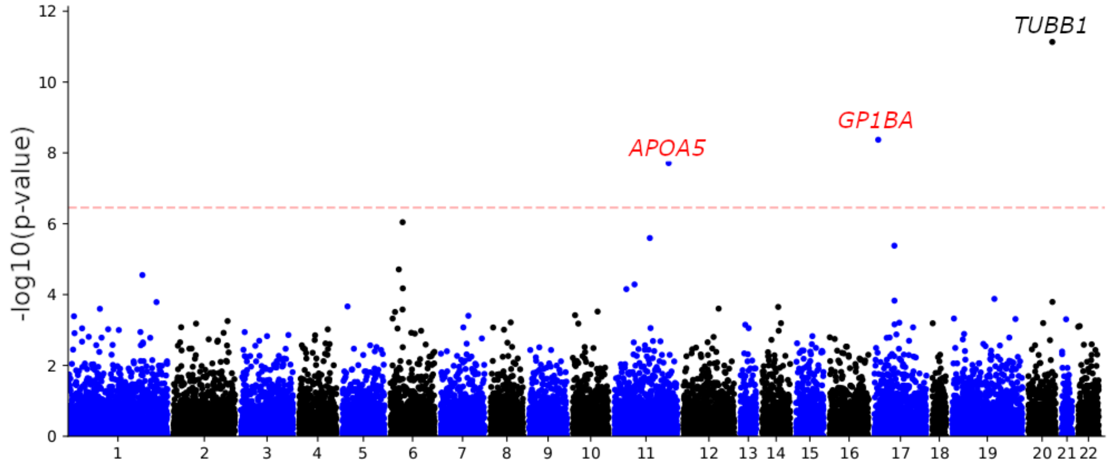

Supplementary Fig. 23. LoF-segment burden exome-wide Manhattan plot for platelet distribution width. Labelled genes are exome-wide significant (after adjusting for multiple testing, $p < 0.05/(14,249 \times 10) = 3.51 \times 10^{-7}$; dashed red line). The LoF-segment burden analysis (with SNP adjustment) used 303,125 UK Biobank samples not included in the exome sequencing cohort. We identified one locus previously reported by Van Hout et al. [8], *TUBB1* (black label), and two additional genes, *APOA5* and *GP1BA* (labels in red). Exact p-values are reported in Supplementary Table 7.

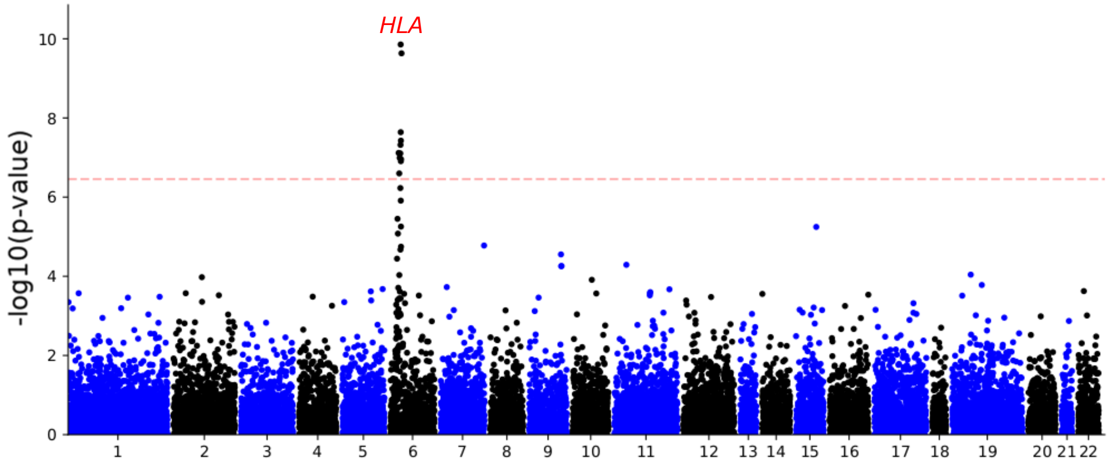

Supplementary Fig. 24. LoF-segment burden exome-wide Manhattan plot for red blood cell count. Labelled genes are exome-wide significant (after adjusting for multiple testing, $p < 0.05/(14,249 \times 10) = 3.51 \times 10^{-7}$; dashed red line). The LoF-segment burden analysis (with SNP adjustment) used 303,125 UK Biobank samples not included in the exome sequencing cohort. We detected one novel association at the HLA locus which was not detected by either of the WES-burden tests. Exact p-values are reported in Supplementary Table 7.

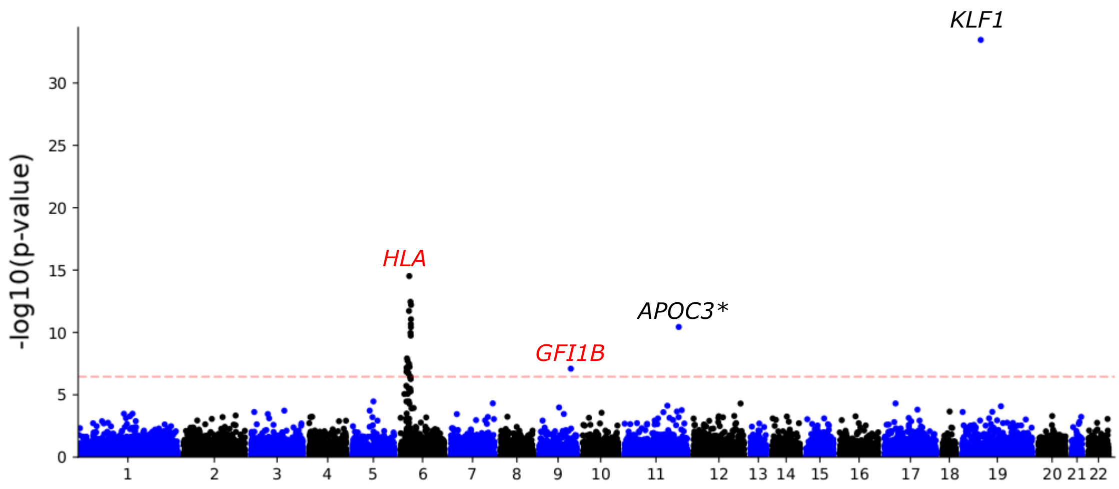

Supplementary Fig. 25. LoF-segment burden exome-wide Manhattan plot for red blood cell distribution width. Labelled genes are exome-wide significant (after adjusting for multiple testing, $p < 0.05/(14,249 \times 10) = 3.51 \times 10^{-7}$; dashed red line). The LoF-segment burden analysis (with SNP adjustment) used 303, 125 UK Biobank samples not included in the exome sequencing cohort. We identified two previously-reported loci (black labels), *KLF1* (detected by Van Hout et al. [8]) and *APOC3* (detected by our WES burden analysis), along with two additional (labels in red). Exact p-values are reported in Supplementary Table 7.

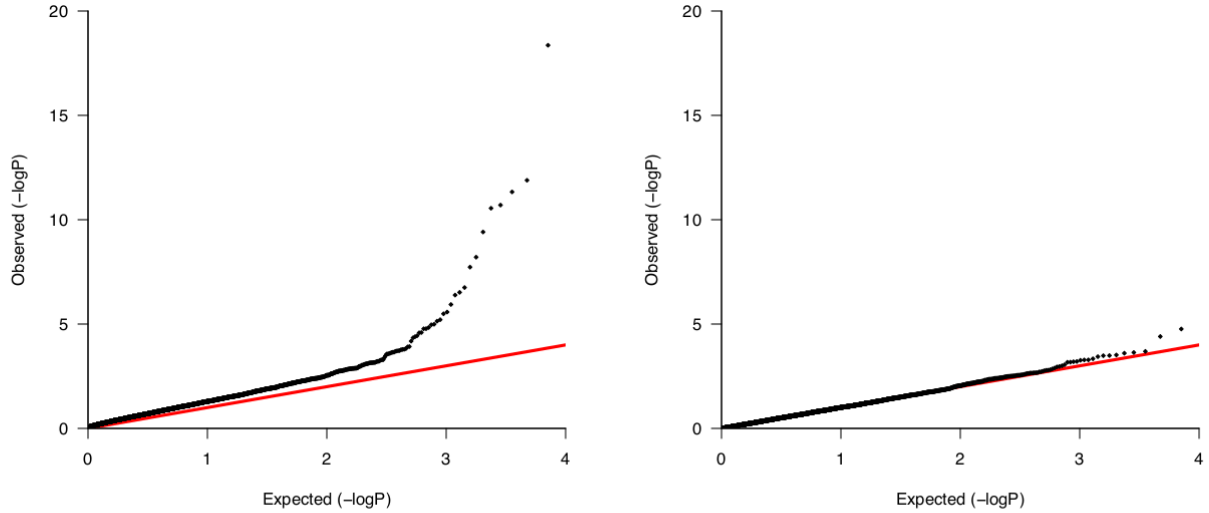

Supplementary Fig. 26. Quantile-quantile plots for LoF-segment burden association. Quantile-quantile plots for mean platelet (thrombocyte) volume (left) and for the same trait, but with randomly permuted phenotype values (right). We observe no signal in the permuted phenotype analysis, suggesting a well-calibrated test. The genomic inflation factor for the (SNP-adjusted) LoF-segment burden test, calculated as the the ratio between the observed median chi-squared association statistic and the median chi-squared association statistic expected under the null, is 1.982 (similar values were observed for the other traits). Values larger than 1 may be caused by pervasive polygenicity [9] and are also observed in analyses of common variants (1.492 for this trait using summary statistics from Loh et al. [10]) and by population stratification remaining after correcting for principal components [11].

Supplementary Tables

Method \ Time		25	50	100	150	200
FastSMC	t	25	50	100	150	200
	length	1.5	1	0.5	0.25	0.1
GERMLINE	min	0.001	0.001	0.001	0.001	0.001
	bits	64	64	64	64	64
	err	0	0	0	0	0
RaPID	l	0.5	0.5	0.5	0.5	0.5
	r	10	10	10	10	10
	s	8	8	8	5	5
	w	3	3	3	3	3
RefinedIBD	length	1.5	1	0.75	0.5	0.1
	lod	1	1	1	1	1

Supplementary Table 1. Optimal parameters from the grid search for IBD detection methods. The accuracy of each method (FastSMC, GERMLINE, RaPID and RefinedIBD) was optimised at different time scale (25, 50, 100, 150 and 200 generations) on a simulated dataset of 300 haploid individuals from a European demographic model and a region of 30 Mb from chromosome 2. FastSMC has two main parameters that we tuned: a time threshold (t) to indicate how deep in time the user wants to detect common ancestry, and a minimum length parameter (in cM) (length) for the IBD segments. The time threshold parameter was always set to be the same as the time threshold used for the benchmarking. We tuned three parameters in GERMLINE: the minimum length of IBD segments (in cM) (min, optimized over $[0.001, 0.01, 0.1, 0.5, 0.75, 1, 1.5, 3, 5]$), the numbers of bits (SNPs) in each window (bits, optimized over $[32, 64, 128, 256]$), the minimum number of mismatches allowed in each of them (err, optimized over $[0, 2, 5, 10, 20]$). RaPID has four major parameters: the minimum IBD segments length (in cM) (l, optimized over $[0.001, 0.01, 0.1, 0.5, 1, 1.5, 3, 5]$), the number of iterations for the PBWT algorithm (r, optimized over $[1, 5, 10, 40, 70]$), the minimum number of successes (s, optimized over $[1, 5, 8, 10, 20, 35, 40, 50, 70]$) and the window size (in SNPs) (w, optimized over $[1, 3, 5, 10, 15, 20, 50, 85, 110, 150]$). RefinedIBD's parameters include a minimum length (in cM) (length, optimized over $[0.001, 0.01, 0.1, 0.5, 0.75, 1, 1.5, 3, 5]$) and a minimum LOD score (proxy for quality) (lod, optimized over $[0.01, 0.1, 1, 3, 5]$). We used default values for all parameters not mentioned here.

time threshold (generations)	average common recall range	method	auPRC	average percent auPRC improvement: 100 ($auPRC_{FastSMC}/auPRC_X$ - 1)
25	[0.07, 0.86]	FastSMC	0.62 (0.06)	
		RefinedIBD	0.54 (0.07)	13.98 (5.70)
		GERMLINE	0.59 (0.06)	4.71 (2.52)
		RaPID	0.46 (0.07)	35.61 (13.23)
50	[0.03, 0.77]	FastSMC	0.59 (0.03)	
		RefinedIBD	0.52 (0.04)	14.61 (4.45)
		GERMLINE	0.57 (0.03)	4.30 (0.88)
		RaPID	0.41 (0.05)	46.27 (12.37))
100	[0.01, 0.66]	FastSMC	0.51 (0.02)	
		RefinedIBD	0.46 (0.03)	10.05 (2.27)
		GERMLINE	0.46 (0.02)	11.34 (1.51)
		RaPID	0.29 (0.04)	80.15 (19.62)
150	[0.01, 0.60]	FastSMC	0.46 (0.01)	
		RefinedIBD	0.44 (0.02)	4.91 (1.54)
		GERMLINE	0.38 (0.01)	19.04 (1.96)
		RaPID	0.22 (0.03)	114.36 (28.98)
200	[0.00, 0.53]	FastSMC	0.41 (0.01)	
		RefinedIBD	0.40 (0.01)	1.04 (1.15)
		GERMLINE	0.33 (0.01)	24.11 (2.36)
		RaPID	0.18 (0.02)	133.95 (32.67)

Supplementary Table 2. Accuracy measurement. Difference in accuracy between FastSMC and other IBD detection methods within the past 25, 50, 100, 150 and 200 generations. We report the percent improvement for the area under the precision-recall curve (auPRC) of FastSMC over other methods. For all methods, precision can only be estimated within a limited recall range (due mostly to the minimum length parameter) and we only report accuracy measurement on the common recall range. Optimal parameters from fine-tuning were used (Supplementary Table 1). Accuracy was measured on 10 realistic simulated datasets, all different from the one used for parameters fine-tuning and all consisting of a 30Mb chromosome under European demographic history model for 300 samples, recombination rates from a human chromosome 2 and SNP ascertainment matching UKBB allele frequencies. Numbers in round brackets represent standard errors over 10 simulations.

time threshold (generations)	average recall range	auPRC
25	[0.07, 0.84]	0.62 (0.09)
50	[0.03, 0.72]	0.57 (0.05)
100	[0.01, 0.65]	0.51 (0.03)
150	[0.0, 0.59]	0.45 (0.02)
200	[0.0, 0.55]	0.4 (0.01)

Supplementary Table 3. Effects of demographic model misspecification. We simulated 10 batches of 300 haploid samples from the first 30Mb of a human Chromosome 2 and a constant population size of $10,000$ diploid individuals. We ran FastSMC at different time scales, assuming a European demographic model and we reported the auPRC and the average recall range. Numbers in round brackets represent standard errors. Values are very similar to results obtained on samples simulated with a European demographic model (Supplementary Table 2). Demographic model misspecification does not impact auPRC.

Chr	Region (Mb)	Min. p-value	Top SNP	Candidate gene(s)	Top gene
1	16.56-17.35	1.59e-07	rs142953444	*RSG1, SDHB, CROCC, MFAP2, NBPF1, SZRD1, FBXO42, NECAP2, ATP13A2, FAM231A, FAM231B, FAM231C, SPATA21, RSG1, SDHB, EPHA2, HSPB7, PADI1, PADI2, PADI3, CLCNKA, CLCNKB, C1orf64, FAM131C, ARHGEF19, C1orf134*	*NBPF1*
1	146.79-147.42	3.23e-09	rs35618507	*ACP6, BCL9, GJA5, GJA8, GPR89B, FMO5, CHD1L, GPR89B, NBPF24, PRKAB2, BX842679.1*	***CHD1L***
1	223.71-224.09	1.65e-07	rs73128182	*APN2, CAPN8, TP53BP2, SUSD4, FBXO28, C1orf65, AC138393.1*	***CAPN8***
2	134.57-138.75	1.00e-20	rs80188644	*LCT*, *DARS*, *HNMT*, *MCM6*, *ACMSD*, *CCNT2*, *CXCR4*, *MGAT5*, *UBXN4*, *R3HDM1*, *THSD7B*, *ZRANB3*, *MAP3K19*, *TMEM163*, *RAB3GAP1*, *HNMT*	*LCT*
6	25.15-33.65	1.00e-20	rs116460689	*HLA* region	*HLA* region
10	17.43-18.10	1.37e-09	rs61842124	*MRC1, STAM, PTPLA, MRC1L1, ST8SIA6, TMEM236, VIM, MRC1, TRDMT1, ST8SIA6, SLC39A12*	***MRC1***
11	0.84-1.71	1.00e-20	rs9704919	*MUC* gene family	*MUC2*
16	70.10-72.69	3.40e-09	rs1833931	*HP, FUK, HPR, TAT, AARS, COG4, IL34, IST1, PDPR, AP1G1, WWP2, ZFHX3, CLEC18A CALB2, CHST4, CMTR2, DHODH, DHX38, HYDIN, SF3B3, VAC14, ZNF19, ZNF23, ATXN1L, DDX19A, DDX19B, EXOSC6, FKSG63, MTSS1L, PHLPP2, PMFBP1, TXNL4B, ZNF821, CLEC18C, ST3GAL2, FLJ00418, MARVELD3, AC009060.1, AC010547.9, RP11-529K1.3*	***HYDIN***
16	88.25-88.48	1.83e-07	rs9925058	*MVD, BANP, CYBA, IL17C, ZFPM1, ZC3H18, ZNF469*	***BANP***
17	41.84-44.95	2.67e-11	rs1230396	*GRN, NSF, PPY, PYY, STH, FZD2, GFAP, GJC1, MAPT, MPP2, MPP3, NAGS, NMT1, UBTF, WNT3, ACBD4, ASB16, C1QL1, CRHR1, DBF4B, DCAKD, DUSP3, FMNL1, G6PC3, HDAC5, LSM12, PLCD3, TMUB2, WNT9B, ADAM11, ARL17A, ARL17B, CCDC43, EFTUD2, HEXIM1, HEXIM2, HIGD1B, ITGA2B, KANSL1, KIF18B, SLC4A1, SPPL2C, ATXN7L3, CCDC103, CD300LG, FAM187A, FAM215A, GPATCH8, LRRC37A, PLEKHM1, RUNDC3A, SPATA32, TMEM101, ARHGAP27, C17orf53, FAM171A2, LRRC37A2, SLC25A39, C17orf104, C17orf105, AC003043.1, AC003102.1, RP11-527L4.2, DHX8, ETV4, SOST, GOSR2, MEOX1, RPRML, WNT9B, RP11-156P1.2*	***EFTUD2***
19	11.19-11.56	1.60e-09	rs36005514	*EPOR, LDLR, RGL3, DOCK6, KANK2, RAB3D, SPC24, PRKCSH, SWSAP1, CCDC151, CCDC159, TMEM205, TSPAN16, C19orf80, DKFZP761J1410, ACP5, CNN1, DNM2, CARM1, ECSIT, ELOF1, TMED1, YIPF2, ELAVL3, ZNF627, ZNF653, SMARCA4, C19orf38, C19orf52, CTC-398G3.6*	*LDLR*
19	44.60-45.45	3.23e-12	rs72480795	*PVR, APOE, BCAM, BCL3, CBLC, APOC1, PVRL2, IGSF23, TOMM40, ZNF112, ZNF180, ZNF224, ZNF225, ZNF226, ZNF227, ZNF229, ZNF233, ZNF234, ZNF235, ZNF285, CEACAM16, CEACAM19, CTC-512J12.6 RELB, APOC2, APOC4, NKPD1, ZNF45, CLASRP, CLPTM1, GEMIN7, ZNF155, ZNF221, ZNF222, ZNF223, ZNF230, ZNF283, ZNF284, ZNF296, ZNF404, BLOC1S3, PPP1R37, TRAPPC6A, AC005779.2, APOC4-APOC2 CTB-129P6.11*	***BCAM***

Supplementary Table 4. Genome-wide significant selection loci. We report loci with elevated values of the DRC_{50} statistic (in the past 50 generations) in the UKBB (1-sided test, after adjusting for multiple testing $p < 0.05/52,003 = 9.6 \times 10^{-7}$). The DRC_{50} statistic of recent positive selection was computed using all 487,409 individuals from the UKBB. When multiple candidate genes were found, we only retained the one nearest to the top SNP, referred to as the top gene (i.e with the smallest p-value). Novel genes are denoted in bold.

Chr	Region (Mb)	Min. p-value	Top SNP	Recombination rate at peak	Percentile recombination rate	Marker density at peak	Percentile marker density
1	16.56-17.35	1.59e-07	rs142953444	0.54	0.9804	12	0.9343
1	146.79-147.42	3.23e-09	rs35618507	0.17	0.687	36	0.2345
1	223.71-224.09	1.65e-07	rs73128182	0.19	0.7286	39	0.1857
2	134.57-138.75	1.00e-20	rs80188644	0.1	0.5096	22	0.5945
6	25.15-33.65	1.00e-20	rs116460689	0.25	0.8159	47	0.0966
10	17.43-18.10	1.37e-09	rs61842124	0.1	0.5327	8	0.9853
11	0.84-1.71	1.00e-20	rs9704919	0.3	0.8655	133	0.003
16	70.10-72.69	3.40e-09	rs1833931	0.45	0.9577	19	0.7041
16	88.25-88.48	1.83e-07	rs9925058	0.19	0.7293	21	0.6344
17	41.84-44.95	2.67e-11	rs1230396	0.04	0.2594	21	0.6338
19	11.19-11.56	1.60e-09	rs36005514	0.44	0.9542	577	0.0001
19	44.60-45.45	3.23e-12	rs72480795	0.45	0.9581	58	0.042

Supplementary Table 5. Marker density and recombination rate percentiles for genome-wide significant selection loci under selection. We divided the genome into 0.1 Mb windows and computed recombination rate and marker density within each window. We then ranked the windows by marker density (from high to low values) and by recombination rate (from low to high values) to get percentiles for each window. We associated each of the selection peaks reported in Supplementary Table 4 to the window they fell in. We report the marker density and recombination rate percentiles for each selection peak.

	LoF	Non-LoF	All
Total number of variants	214,753	10,233,971	10,448,724
Number of age-estimated variants	3,445	159,614	163,059
Mean age estimate	6,377.95	6,487.54	6,485.22
Median age estimate	879.4	904.86	904.25
95% age estimates CI	$[106.57; 47,794.92]$	$[116.62; 48,002.80]$	$[116.51; 48,002.80]$

Supplementary Table 6. Age estimates for exome sequencing alleles. We report a summary of the allele age estimates (in generations) for a subset of exome sequencing variants analyzed by Albers et al. [12]. All corresponds to the complete set of exome-sequenced variants; LoF refers to loss-of-function mutations we analyzed and non-LoF refers to its complement.

Trait	Gene	LoF-segment burden not SNP-adjusted	LoF-segment burden SNP-adjusted	WES LoF burden not SNP-adjusted	WES LoF burden SNP-adjusted
Eosinophil count	***IL33***	2.19E-18	8.64E-15	2.01E-03	6.85E-03
	HIST1H1A	4.17E-16	1.62E-07	6.78E-01	6.81E-01
	HIST1H1C	4.67E-19	8.25E-09	2.94E-01	2.80E-01
	HIST1H1T	5.17E-15	2.67E-07	9.48E-01	9.58E-01
	HIST1H2BF	1.05E-13	3.31E-07	2.65E-03	2.77E-03
	HIST1H3E	1.76E-17	1.94E-08	1.46E-01	1.48E-01
	HIST1H4F	1.39E-18	2.35E-09	7.66E-01	7.71E-01
	BTN3A2	1.23E-17	2.32E-09	3.56E-01	3.56E-01
	BTN2A2	8.02E-21	7.20E-11	5.59E-01	5.63E-01
	BTN3A3	5.36E-17	3.80E-09	9.55E-01	9.65E-01
	BTN2A1	1.65E-17	6.95E-10	9.75E-01	1.00E+00
	BTN1A1	4.33E-13	2.06E-07	1.00E+00	9.92E-01
	ABT1	4.12E-18	3.55E-10	2.15E-01	2.18E-01
	HIST1H2AG	2.71E-16	5.81E-15	4.09E-02	4.13E-02
	HIST1H2AH	3.72E-19	1.51E-17	2.75E-01	2.73E-01
	PRSS16	1.66E-12	1.75E-11	6.60E-02	6.62E-02
	POM121L2	1.28E-18	2.98E-17	6.73E-01	6.76E-01
	ZNF391	4.30E-26	1.74E-24	4.71E-01	4.69E-01
	HIST1H2BM	4.32E-17	4.01E-16	3.29E-02	3.25E-02
	HIST1H2AK	8.31E-11	9.96E-10	1.73E-01	1.71E-01
	HIST1H2BO	2.90E-10	1.61E-09	7.40E-01	7.42E-01
	OR2B2	1.36E-23	5.80E-22	4.20E-02	4.27E-02
	OR2B6	2.21E-08	2.01E-07	6.87E-02	6.84E-02
	ZNF165	3.50E-22	1.48E-20	9.55E-01	9.36E-01
	ZSCAN16	2.52E-21	5.39E-20	2.41E-01	2.41E-01
	ZKSCAN8	2.26E-07	2.96E-07	6.54E-01	6.40E-01
	ZSCAN9	2.89E-13	9.59E-12	3.44E-01	3.40E-01
	ZKSCAN4	1.14E-15	2.63E-14	4.15E-01	4.18E-01
	NKAPL	9.19E-16	4.63E-15	5.71E-01	5.54E-01
	PGBD1	5.04E-24	1.99E-22	1.06E-01	1.06E-01
	ZSCAN31	1.12E-28	1.21E-26	4.16E-01	4.31E-01
	ZSCAN12	5.12E-20	1.43E-18	4.51E-01	4.55E-01
	ZSCAN23	7.33E-10	3.59E-09	5.07E-01	5.07E-01
	GPX6	5.40E-17	8.29E-16	3.86E-01	3.89E-01
	PSORS1C2	8.36E-26	3.71E-22	5.32E-01	5.43E-01
	RPH3A	2.27E-07	7.63E-14	3.16E-01	2.74E-01
	OAS3	5.49E-04	2.76E-08	2.07E-02	1.63E-02
	GFI1B	1.93E-07	1.92E-07	3.96E-01	3.99E-01
Mean corpuscular haemogliobin	***KLF1***	1.88E-20	6.79E-21	9.11E-15	1.51E-14
	GMPR	6.60E-12	7.60E-11	2.94E-06	7.73E-06
	HIST1H2BA	6.40E-31	9.26E-28	5.50E-01	4.65E-01
	SLC17A2	2.58E-03	1.29E-08	5.34E-01	5.29E-01
	HIST1H2AB	2.13E-20	5.30E-21	9.81E-01	9.13E-01
	HFE	1.39E-02	6.90E-10	5.68E-01	5.44E-01
	HIST1H4C	1.60E-16	2.13E-26	3.33E-01	3.47E-01
	HIST1H2BD	2.86E-05	4.24E-10	7.62E-01	8.38E-01
	HIST1H4D	4.02E-69	3.82E-69	7.34E-01	7.21E-01
	HIST1H2BG	2.89E-19	9.86E-17	5.73E-01	5.13E-01
	HIST1H2AE	1.68E-12	5.62E-08	6.05E-01	7.18E-01
	HIST1H1D	1.28E-05	6.79E-15	6.73E-01	6.17E-01
	BTN2A1	1.98E-07	2.07E-19	3.18E-01	2.97E-01
	HIST1H2AG	6.61E-11	6.77E-08	3.86E-01	2.63E-01
	HIST1H4I	2.01E-17	1.42E-22	4.39E-02	3.64E-02
	ZNF184	3.95E-33	1.74E-07	1.48E-01	1.66E-01
	CHEK2	5.58E-08	1.43E-07	1.44E-04	2.19E-04
	PSORS1C2	2.49E-17	1.47E-17	9.16E-01	9.23E-01
Mean platelet thrombocyte volume	***GP1BA***	3.48E-20	1.82E-19	8.84E-08	1.03E-07
	HIST1H4D	2.54E-07	7.26E-08	1.84E-01	2.10E-01
	POM121L2	2.39E-06	1.46E-08	2.73E-01	2.84E-01
	OR2B6	6.48E-08	8.39E-08	3.69E-01	3.64E-01
	CHEK2	5.98E-08	1.93E-07	1.21E-01	1.38E-01
	POLK	2.01E-11	4.17E-09	5.93E-01	6.37E-01
	IQGAP2	4.49E-36	4.40E-34	3.72E-15	2.55E-15
	CENPBD1	1.64E-07	2.61E-07	2.67E-01	2.74E-01
	MLXIP	4.23E-10	6.29E-10	2.31E-03	1.12E-03
	KALRN	2.31E-14	3.79E-12	3.85E-18	3.01E-17
	ZNF664	2.11E-09	3.98E-08	7.75E-01	7.94E-01
	OR6F1	7.01E-12	1.44E-08	4.78E-01	7.77E-01
Platelet count	*GP1BA*	5.97E-08	1.43E-07	3.59E-05	4.47E-05
	ABT1	7.91E-02	1.10E-07	4.78E-03	5.28E-03
	MPL	3.08E-07	1.99E-07	1.86E-04	1.54E-04
	IQGAP2	5.26E-08	6.52E-08	1.72E-04	1.13E-04
	KCTD10	1.49E-10	2.11E-07	1.30E-02	1.28E-02
	TCHP	2.76E-13	1.73E-11	3.98E-01	3.73E-01
	RPH3A	4.06E-08	4.82E-13	9.14E-01	9.57E-01

Trait	Gene	LoF-segment burden not SNP-adjusted	LoF-segment burden SNP-adjusted	WES LoF burden not SNP-adjusted	WES LoF burden SNP-adjusted
Platelet distribution width	*GP1BA*	7.51E-10	4.26E-09	7.37E-06	9.78E-06
	TUBB1	6.10E-12	7.38E-12	7.34E-18	1.23E-17
	APOA5	1.12E-08	1.94E-08	2.14E-01	2.39E-01
Red blood cell count	*BTN2A1*	7.79E-11	7.65E-08	5.42E-01	5.39E-01
	POM121L2	6.22E-08	2.52E-07	2.56E-01	2.53E-01
	HIST1H2BM	8.71E-08	1.02E-07	4.18E-01	4.17E-01
	HIST1H2BO	2.79E-08	7.86E-08	1.70E-01	1.65E-01
	ZNF165	3.54E-08	4.79E-08	8.95E-01	9.59E-01
	ZSCAN9	4.04E-11	1.39E-10	5.86E-01	5.75E-01
	ZKSCAN4	9.64E-09	2.29E-08	5.24E-01	5.04E-01
	PGBD1	1.05E-07	1.24E-07	6.66E-01	7.48E-01
	ZSCAN31	3.36E-08	3.75E-08	7.18E-01	7.53E-01
	GPX6	7.71E-07	1.11E-07	4.62E-01	4.64E-01
	PSORS1C2	3.16E-11	2.33E-10	1.61E-01	1.55E-01
Red blood cell distribution width	***KLF1***	4.60E-33	3.49E-34	6.95E-13	5.69E-13
	HIST1H1A	1.48E-27	1.42E-07	9.81E-01	9.87E-01
	HIST1H3A	7.40E-28	6.91E-08	2.60E-01	2.90E-01
	HIST1H1C	2.01E-30	1.21E-08	9.70E-01	9.66E-01
	HIST1H1T	1.29E-25	6.54E-08	3.33E-01	4.61E-01
	HIST1H4D	1.04E-33	1.86E-08	8.85E-01	8.07E-01
	HIST1H4F	9.32E-25	1.60E-07	1.54E-01	1.87E-01
	BTN3A2	4.12E-23	1.95E-07	7.95E-01	7.46E-01
	HIST1H2AG	3.48E-17	1.90E-12	4.03E-01	3.08E-01
	HIST1H2AH	1.07E-14	3.34E-08	8.55E-01	7.89E-01
	ZNF391	1.44E-20	3.03E-15	2.88E-01	3.13E-01
	HIST1H2BM	2.06E-13	5.75E-08	7.22E-01	7.06E-01
	HIST1H2AM	1.79E-09	3.36E-07	3.34E-01	3.26E-01
	ZNF165	1.35E-14	3.51E-13	7.91E-01	7.54E-01
	ZSCAN16	7.53E-12	1.09E-10	5.50E-01	5.76E-01
	NKAPL	1.11E-13	1.81E-10	8.64E-01	8.35E-01
	PGBD1	1.58E-12	2.18E-11	4.90E-01	4.88E-01
	ZSCAN31	1.06E-12	3.77E-11	6.55E-01	5.65E-01
	ZSCAN12	1.22E-17	8.79E-12	4.83E-01	4.22E-01
	GPX6	8.37E-19	6.20E-13	4.76E-01	4.89E-01
	APOC3	1.37E-12	3.67E-11	2.13E-07	2.57E-07
	GFI1B	3.93E-07	8.10E-08	6.32E-01	6.40E-01

Supplementary Table 7. Exome-wide significant genes by LoF-segment burden, and comparison to other tests. A detailed comparison between the four association tests we performed. We report all genes found significant using SNP-adjusted LoF-segment burden (using 303, 125 non-sequenced samples; 111 associations in total), as well corresponding p-values for LoF-segment burden without SNP-adjustment, and WES-LoF association (using 34, 422 sequenced samples) with or without SNP-adjustment. P-values are computed using two-sided t-tests. Genes in bold correspond to hits previously reported by Van Hout et al. [8], for a total of eight replicated signals at exome-wide significance in the non-sequenced cohort. SNP-adjustments resulted in decreased significance for the LoF-segment burden test, but had a smaller effect on the WES-LoF-based approach; in total WES-LoF with SNP-adjustment achieved similar p-values in all but one (*GMPR*) of the exome-wide significant hits obtained by the simple WES-LoF test.

	Gene	Trait	WES LoF burden
1	*TET2*	Eosinophil count	1.79E-08
2	*GMPR*	Mean corpuscular haemoglobin	2.94E-06
3	*KLF1*	Mean corpuscular haemoglobin	9.11E-15
4	*TUBB1*	Mean platelet (thrombocyte) volume	3.01E-07
5	*GP1BA*	Mean platelet (thrombocyte) volume	8.84E-08
6	*IQGAP2*	Mean platelet (thrombocyte) volume	3.72E-15
7	*KALRN*	Mean platelet (thrombocyte) volume	3.85E-18
8	*TUBB1*	Platelet count	7.45E-07
9	*ASXL1*	Platelet distribution width	1.44E-06
10	*TUBB1*	Platelet distribution width	7.34E-18
11	*MAPK8*	Red blood cell distribution width	1.33E-06
12	*APOC3*	Red blood cell distribution width	2.13E-07
13	*KLF1*	Red blood cell distribution width	6.95E-13

Supplementary Table 8. Exome-wide significant genes by our WES-LoF Burden. Association statistics for the 13 significant loci detected by our WES-LoF burden test (not SNP-adjusted), similar to Van Hout et al. [8] but using a simple linear model and $34,422$ exome-sequenced samples. P-values are computed using two-sided t-tests.

	Gene	Trait	WES LoF burden p	WES LoF burden R^2	LoF-segment burden p	LoF-segment burden R^2	R^2_{prop} (%)
1	*IL33*	Eosinophil count	2.01E-03	2.85E-04	8.64E-15	2.06E-04	72.26
2	*GP1BA*	Mean platelet thrombocyte volume	8.84E-08	8.51E-04	1.82E-19	2.77E-04	32.57
3	*TUBB1*	Platelet distribution width	7.34E-18	2.20E-03	7.38E-12	1.60E-04	7.25
4	*TUBB1*	Mean platelet thrombocyte volume	3.01E-07	7.81E-04	2.15E-03	3.21E-05	4.11
5	*TUBB1*	Platelet count	7.45E-07	7.29E-04	4.21E-05	5.71E-05	7.84
6	*HBB*	Red blood cell distribution width	3.49E-02	1.32E-04	2.25E-03	3.18E-05	23.99
7	*HBB*	Red blood cell count	7.95E-02	9.15E-05	2.68E-02	1.67E-05	18.23
8	*KLF1*	Red blood cell distribution width	6.95E-13	1.53E-03	3.49E-34	5.06E-04	32.99
9	*KLF1*	Mean corpuscular haemoglobin	9.11E-15	1.79E-03	6.79E-21	2.99E-04	16.76
10	*ASXL1*	Platelet distribution width	1.44E-06	6.91E-04	1.58E-01	6.78E-06	0.98
11	*ASXL1*	Red blood cell distribution width	8.23E-04	3.33E-04	3.15E-01	3.43E-06	1.03
12	*KALRN*	Mean platelet thrombocyte volume	3.85E-18	2.24E-03	3.79E-12	1.64E-04	7.33
13	*IQGAP2*	Mean platelet thrombocyte volume	3.72E-15	1.84E-03	4.40E-34	5.04E-04	27.43
14	*GMPR*	Mean corpuscular haemoglobin	2.94E-06	6.50E-04	7.60E-11	1.44E-04	22.18

Supplementary Table 9. Comparison between association analyses. Association statistics for the 14 significant loci detected by Van Hout et al. [8] reported in Table 1, here including the p-value and the phenotypic variance explained (R^2) by each approach. The last column reports the estimated proportion of the phenotypic variation (R^2_{prop}, in %) of the sequenced samples that can be explained by the non-sequenced cohort; on average that is 19.64% for all the 14 reported associations, or 27.35% when restricting to the exome-wide significant signals.

Supplementary References

[1] Browning, B. L. & Browning, S. R. Improving the accuracy and efficiency of identity-by-descent detection in population data. *Genetics* **194**, 459–471 (2013). URL `https://www.genetics.org/content/194/2/459`.

[2] Gusev, A. *et al.* Whole population, genomewide mapping of hidden relatedness. *Genome research* **19**, 318–26 (2008).

[3] Naseri, A., Liu, X., Tang, K., Zhang, S. & Zhi, D. RaPID: ultra-fast, powerful, and accurate detection of segments identical by descent (IBD) in biobank-scale cohorts. *Genome Biology* **20** (2019).

[4] Bycroft, C. *et al.* The UK Biobank resource with deep phenotyping and genomic data. *Nature* **562**, 203 (2018).

[5] Umeyama, S. Least-squares estimation of transformation parameters between two point patterns. *IEEE Transactions on Pattern Analysis and Machine Intelligence* **13**, 376–380 (1991).

[6] Gusev, A. *et al.* The architecture of long-range haplotypes shared within and across populations. *Molecular biology and evolution* **29**, 473–486 (2011).

[7] Manichaikul, A. *et al.* Robust relationship inference in genome-wide association studies. *Bioinformatics* **26**, 2867–2873 (2010).

[8] Van Hout, C. V. *et al.* Whole exome sequencing and characterization of coding variation in 49,960 individuals in the UK Biobank. Preprint at `https://www.biorxiv.org/content/10.1101/572347v1` (2019).

[9] Yang, J. *et al.* Genomic inflation factors under polygenic inheritance. *European Journal of Human Genetics* **19**, 807–812 (2011).

[10] Loh, P.-R., Kichaev, G., Gazal, S., Schoech, A. P. & Price, A. L. Mixed-model association for biobank-scale datasets. *Nature Genetics* **50**, 906 (2018).

[11] Mathieson, I. & McVean, G. Differential confounding of rare and common variants in spatially structured populations. *Nature genetics* **44**, 243 (2012).

[12] Albers, P. K. & McVean, G. Dating genomic variants and shared ancestry in population-scale sequencing data. *PLoS biology* **18**, e3000586 (2020).
